# Nuciferine reduces vascular leakage and improves cardiac function in acute myocardial infarction by regulating the PI3K/AKT pathway

**DOI:** 10.1038/s41598-024-57595-w

**Published:** 2024-03-26

**Authors:** Wei Xie, Shumin Chen, Wenzhe Wang, Xichun Qin, Chuiyu Kong, Dongjin Wang

**Affiliations:** 1 Department of Cardio-Thoracic Surgery, Nanjing Drum Tower Hospital, The Affiliated Hospital of Nanjing University Medical School, Nanjing, 210008 Jiangsu China; 2 Department of Critical Care Medicine, Nanjing Drum Tower Hospital, The Affiliated Hospital of Nanjing University Medical School, Nanjing, Jiangsu China; 3 Institute of Cardiothoracic Vascular Disease, Nanjing University, Nanjing, Jiangsu China

**Keywords:** Nuciferine, AMI, Endothelial barrier, PI3K/AKT pathway, Myocardial infarction, Cell biology, Drug discovery

## Abstract

The destruction of the microvascular structure and function can seriously affect the survival and prognosis of patients with acute myocardial infarction (AMI). Nuciferine has a potentially beneficial effect in the treatment of cardiovascular disease, albeit its role in microvascular structure and function during AMI remains unclear. This study aimed to investigate the protective effect and the related mechanisms of nuciferine in microvascular injury during AMI. Cardiac functions and pathological examination were conducted in vivo to investigate the effect of nuciferine on AMI. The effect of nuciferine on permeability and adherens junctions in endothelial cells was evaluated in vitro, and the phosphorylation level of the PI3K/AKT pathway (in the presence or absence of PI3K inhibitors) was also analyzed. In vivo results indicated that nuciferine inhibited ischemia-induced cardiomyocyte damage and vascular leakage and improved cardiac function. In addition, the in vitro results revealed that nuciferine could effectively inhibit oxygen–glucose deprivation (OGD) stimulated breakdown of the structure and function of human coronary microvascular endothelial cells (HCMECs). Moreover, nuciferine could significantly increase the phosphorylation level of the PI3K/AKT pathway. Finally, the inhibitor wortmannin could reverse the protective effect of nuciferine on HCMECs. Nuciferine inhibited AMI-induced microvascular injury by regulating the PI3K/AKT pathway and protecting the endothelial barrier function in mice.

## Introduction

Cardiovascular diseases are the foremost cause of morbidity, disability, and death worldwide^1,2^. They impose heavy costs on health systems and society, including costs incurred due to productivity and economic losses^3^. Acute myocardial infarction (AMI) is among the most dangerous cardiovascular diseases. With the continuous advancement of treatments, morbidity, and mortality associated with AMI have recently decreased significantly^4^. However, AMI is a huge burden on the patient's life and is associated with complications such as arrhythmia, shock, and heart failure in the later stages^5^.

In addition to protecting damaged cardiomyocytes as much as possible, microvascular, structural, and functional integrity is also a key prognostic factor^6^. During AMI treatment, even if infarct-related vessels are successfully opened, an ischemic injury will be further aggravated after perfusion based on pathological changes in microvessels before recanalization^7^. Receiving a completely effective blood supply is difficult for the myocardium. The American College of Cardiology/American Heart Association guidelines recommend minimization of microvascular damage as the priority strategy for post-AMI management^8^. Novel drugs must be developed to maintain the stability of the microvascular structure and function, and they should be administered to AMI patients conveniently and efficiently. This can provide a stable internal environment basis for efficient coronary artery recanalization in the later stage, as well as for post-recanalization cardiac perfusion and myocardial repair. However, how to maintain the stability of the microvascular structure and function in AMI patients has always been a scientific and technical challenge.

Researchers have recently focused more on natural medicines because of their protective effects in managing and preventing oxidative stress or inflammation-related diseases and because of their fewer side effects^9^. Many biologically active plant natural products have received considerable attention because of their therapeutic effects on inflammatory diseases resulting from their unique pharmacological properties and low toxicity^10^. Nuciferine is a major bioactive ingredient extracted from the lotus leaf. It has various pharmacological effects such as anticancer, anti-inflammatory, and antioxidant effects^11,12^. In addition, nuciferine alleviates myocardial cell apoptosis caused by AMI and ischemia–reperfusion through anti-inflammatory and antioxidant stress^13,14^.

To date, the effects of nuciferine on the microvascular structure and function during AMI, and the related signaling pathways mediated by this drug during acute ischemia and hypoxia remain unknown. Therefore, the protective effect of nuciferine on the microvascular structure and function was investigated here in mouse AMI models. We also elucidated the potential mechanism through which nuciferine mediates the endothelial barrier protective function.

## Materials and methods

### Mice and mouse AMI model

All animal experiments were conducted according to the Model Animal Research Center of Nanjing University. The experimental protocol was approved by the Animal Protection and Use Committee of Nanjing University School of Medicine. C57BL/6J male mice (weight: 20 ± 3 g, age: 8 weeks) were reared in a specific pathogen-free environment. After being domesticated for a week, the mice were used to establish AMI models. Briefly, the mice were anesthetized by intraperitoneally injecting pentobarbital sodium (45 mg/kg). The heart was then exposed through an incision in the left chest, and a 7.0 slip-suture slip knot was ligated around the left anterior descending coronary artery (LAD) to induce MI for 6 h. The mice in the sham and sham treatment (nuciferine, 10 mg/kg) groups underwent surgery to expose the heart but had no LAD ligature. The treatment group was intragastrically administered 10 mg/kg of nuciferine 4 h before establishing the model^14,15^.

### Myocardial Evans Blue/TTC staining

Before obtaining the mouse heart, 1 mL of 0.5% Evans Blue dye (E2129, Sigma) was injected along the ascending aorta. The mouse hearts were refrigerated at − 20 °C for 20 min and cut into 2-mm-thick slices perpendicular to the left anterior descending branch. The sample was then immersed in 1% of 2,3,5-triphenyltetrazolium (T8877, Sigma) and incubated at 37 °C for 10–15 min to distinguish between the infarcted tissue and the live myocardial muscle. The white tissue represents the infarct area, the red tissue represents the ischemic area, and the black and blue tissue represents the normal myocardial tissue. All partial area statistics are captured using ImageJ.

### Cardiac function evaluation

The left ventricular ejection fraction (LEF) was measured through 2-D guided M-mode echocardiography. The left ventricular end-diastolic volume (EDV) and end-systolic volume (ESV) were measured using Vevo2100 (Japan). LEF is calculated as EF = (EDV − ESV)/EDV × 100%. All measurements were based on an average of at least three cardiac cycles.

### Detection of cardiac troponin-T (cTnT), TNF-α, and IL-6

The cTnT, TNF-α, and IL-6 levels were examined using quantitative ELISA kits (Shanghai Renjie Biotechnology Co., Ltd.) according to the kit instructions. After the model of each group was established successfully, 0.5 mL venous blood was collected for cTnT concentration detection. Similarly, the myocardial tissue of the infarct area and the corresponding part of the myocardial tissue of control mice was homogenized, and the supernatant was used for detecting TNF-α and IL-6 concentrations.

### Immunofluorescence staining

Myocardial or cell samples were immobilized with 4% paraformaldehyde for 15 min, permeated with Triton X-100 (0.1%), sealed with a solution containing 5% bovine serum for 1 h, and treated with a primary antibody. The samples were then incubated with anti-mouse CD31 (ab9498, Abcam), anti-rabbit fibronectin (15613-1-AP, Proteintech), and anti-rabbit VE-cadherin (ab205336, Abcam) antibodies for 12 h at 4 °C. The samples were then incubated with the secondary antibody for 1 h at room temperature in the dark. DAPI (MBD0015, Sigma) was used to stain nuclei. After the samples were washed with PBS, they were observed under the fluorescence microscope (Olympus). All partial area statistics were captured using ImageJ.

### Cell culture and establishment of the oxygen–glucose deprivation model

Human coronary microvascular endothelial cells (HCMECs) were purchased from Bnbio (Beijing, China). The HCMECs were cultured in EC medium (1001, ScienCell) containing 5% fetal bovine serum, 1% endothelial cell growth, and 1% penicillin/streptomycin. To establish an oxygen–glucose deprivation (OGD) model, the medium was changed to glucose-free and serum-free medium and cultured in a three-gas incubator (Heal Force BioMeditech Holdings) with 94% N_2_, 5% CO_2_, and 1% O_2_ for 1, 2, 4, or 8 h. The HCMECs were treated with or without nuciferine (in the presence or absence of PI3K inhibitors, wortmannin, 50 nM). After the OGD model was established, the cells were harvested for subsequent experiments.

### Western blotting analysis

Total protein was extracted from the myocardial tissue in the infarct area and the corresponding surviving myocardial tissue or HCMECs by using RIPA lysis buffer. Membrane and cytosol proteins were extracted according to the manufacturer’s instructions (P0033, Beyotime). The bicinchoninic acid (P0011, Beyotime) kit was used to determine the protein concentration. Then, 10% SDS-PAGE was used to separate the cell or tissue lysate. Once the protein transfer step was completed, the PVDF membrane (ISEQ00010, Millipore) was sealed with 5% skimmed milk and incubated with anti-rabbit VE-cadherin (ab205336, Abcam), anti-rabbit PI3K (4257, Cell Signaling Technology), anti-rabbit Phospho-PI3K (17366, Cell Signaling Technology), anti-rabbit AKT (4691, Cell Signaling Technology), anti-rabbit Phospho-AKT (4060, Cell Signaling Technology), and anti-mouse β-tubulin (66240-1-Ig, Proteintech) antibodies overnight at 4 °C. After the samples were washed three times in tributyltin compound buffer, they were incubated at room temperature for 1 h. Finally, the proteins were visualized using the ECL solution (KGP112, Jiangsu Kechuang Biotechnology Co., LTD.). Blot analysis was performed using the Tanon image system (TANON-1600, Tanon).

### Permeability measurement of HCMECs

The cell culture transwell (0.4 μm, Corning) was used to detect HCMEC permeability. The cells were conjoined on porous polyester film inserts, and 150 and 500 μL ECM were added to the upper and lower chambers, respectively. Dextran-FITC (10 μM) was added to the upper cavity. Following OGD treatment, the fluorescence intensity of the sample was measured by a microplate reader (Multiskan GO, Thermo) through the 96-well cluster plate. Dextran-FITC passing through the endothelial monolayer was normalized to the fluorescence reading of the upper cavity. Permeability was calculated in relative fluorescence units.

### CCK-8 analysis

HCMECs were inoculated into 96-well plates and cultured in cell incubators. Nuciferine of the corresponding concentration was incubated for 24 h according to the experimental requirements. Fresh medium containing the CCK-8 (C0041, Beyotime) reagent (10:1) was added and cultured at 37 °C for another 2 h. The sample was then examined at 450 nm by using a microplate reader (Multiskan GO, Thermo).

### Detection of lactate dehydrogenase cytotoxicity

Lactate dehydrogenase (LDH) was assessed in all experimental groups by using the LDH activity assay kits (C0016, Beyotime).

### Heatmap and KEGG enrichment analysis

The dataset regarding acute myocardial infarction was acquired from the NCBI Gene Expression Omnibus database (GEO; https://www.ncbi.nlm.nih.gov/geo/). The GSE147365 dataset consisted of 6 control samples and 6 heart tissues obtained from mice suffering from acute myocardial infarction. Differential gene expression analysis between the disease and control groups was performed using the "Limma" package^16^, with a significance threshold set at *P* < 0.05 and a fold change greater than 2. Visualization of the obtained results was accomplished through the utilization of heatmap. Additionally, KEGG enrichment analyses were conducted on the identified critical genes associated with the disease, utilizing the "org.Mm.eg.db" and "clusterProfiler" packages^17^. Significance was defined at a threshold of *P* < 0.05.

### Statistical analysis

Data are expressed as mean ± standard deviation. Multiple group comparisons were performed using one-way ANOVA, followed by a minimum significant difference T-test for post hoc analysis. The two-tailed Student's t-test was used to compare data between the two independent groups. SPSS 25 software (Chicago, IL, USA) was used for statistical analysis. *P* < 0.05 was considered statistically significant.

### Ethics approval and consent to participate

The study was conducted in accordance with the ARRIVE guidelines and animal experiments were carried out in accordance with the guide for the Care and Use of Laboratory Animals published by the US National Institutes of Health (NIH Publication, 8th Edition, 2011).

## Results

### Nuciferine reduces myocardial infarct area and vascular endothelial permeability in AMI mice

**Figure 1:**
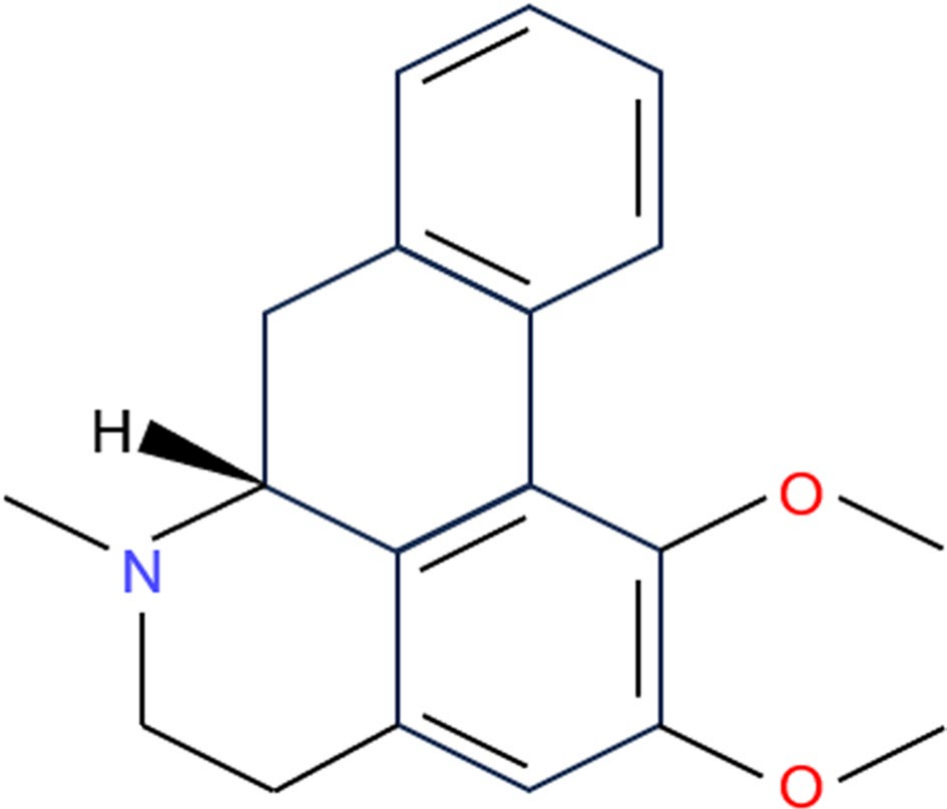
Structural diagram of nuciferine.

**Figure 2:**
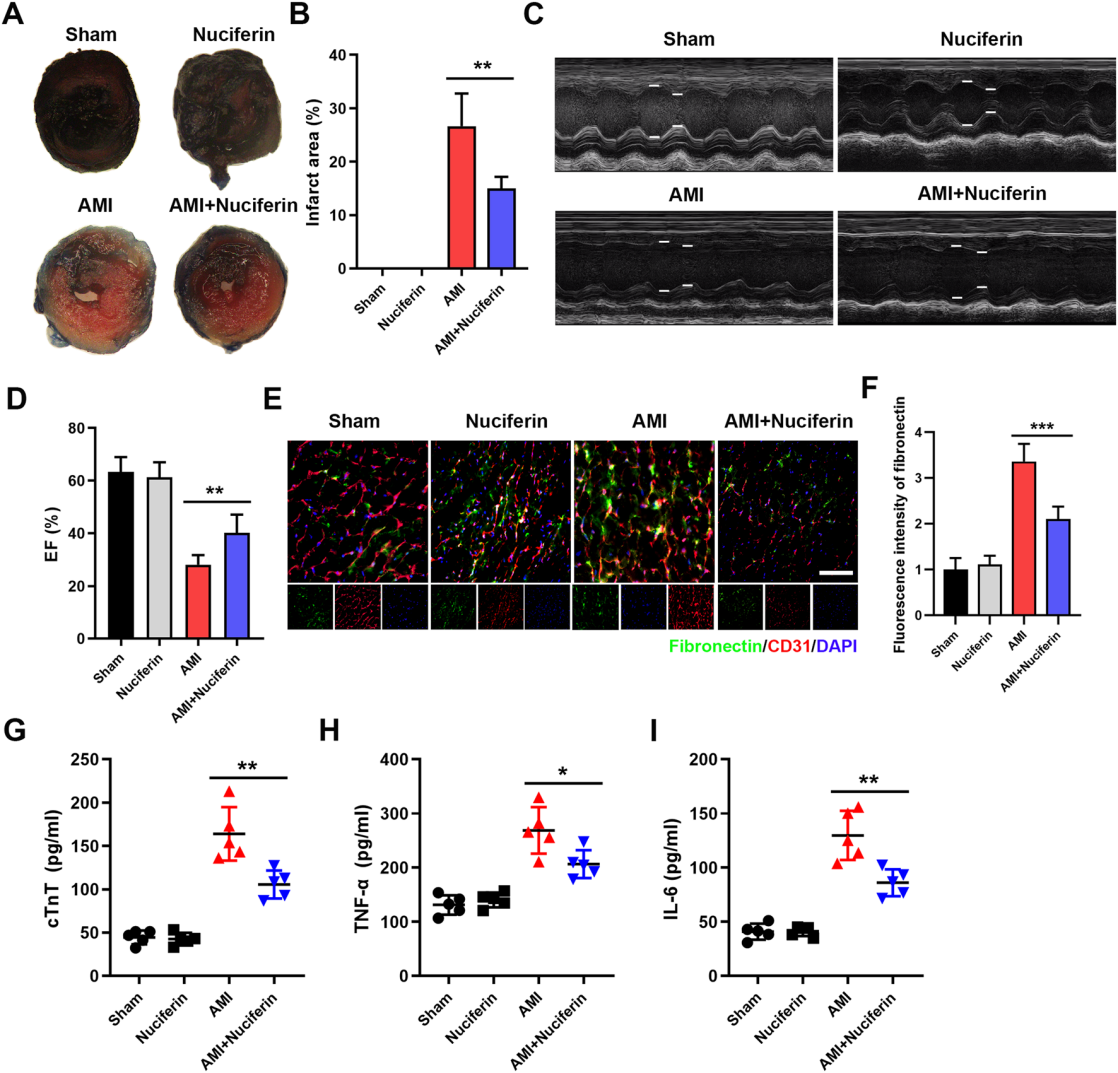
Nuciferine reduces myocardial infarction size and vascular leakage in AMI mice. (**A**) Representative images of Evans blue/TTC staining of the myocardial tissues. The white tissue represents the infarct area, while the red tissue represents the ischemic area, n = 5. (**B**) Quantitative statistics of the infarct size, n = 5. (**C**) Representative images of transthoracic M-mode echocardiography. (**D**) Quantitative statistics of left ventricular ejection fraction, n = 5. (**E**) Immunofluorescence to evaluate the effect of nuciferine on vascular leakage in myocardial tissue of mice, Scale bar = 100 μm, n = 5. (**F**) Quantification of fluorescence intensity of fibronectin, n = 5. (**G**) Quantification of cTnT in the serum of each group, n = 5. Quantification of TNF-α (**H**) and IL-6 (**I**) content in the myocardial tissues of each group, n = 5. Data are expressed as the mean ± SD, *P < 0.05, **P < 0.01, ***P < 0.001 *vs.* indicated group.

To investigate the potential effects of nuciferine (Fig. 1) on the ischemic myocardial endothelial barrier, the mice were intragastrically administered with nuciferine 4 h before establishing the AMI model. Evans Blue/TTC staining was performed to evaluate the MI size. Two-dimensional-guided M-mode echocardiography was performed to determine the LEF. As shown in Fig. 2A–D, AMI results in obvious infarction in the myocardial tissue. Nuciferine can significantly reduce the myocardial infarct size and improve cardiac function in mice. This result suggested that nuciferine significantly reduces myocardial damage in AMI. Subsequently, the degree of fibronectin leakage was detected through immunofluorescence. Significant leakage of myocardial tissue vessels was observed after ischemia, which is partially reversed by nuciferine (Fig. 2E,F). In addition, the expression of cTnT, a marker of myocardial injury^18^, was detected to evaluate the degree of myocardial tissue injury. The ELISA results revealed that nuciferine significantly reduces the serum cTnT concentration in the AMI mice (Fig. 2G). Consistent with this observation, TNF-α and IL-6 concentrations in the myocardium also decreased after nuciferine treatment (Fig. 2H,I). These results suggest that nuciferine positively affects the reduction in the MI size and vascular endothelial permeability after AMI in mice.

### Establishment of the OGD model of endothelial cells

**Figure 3:**
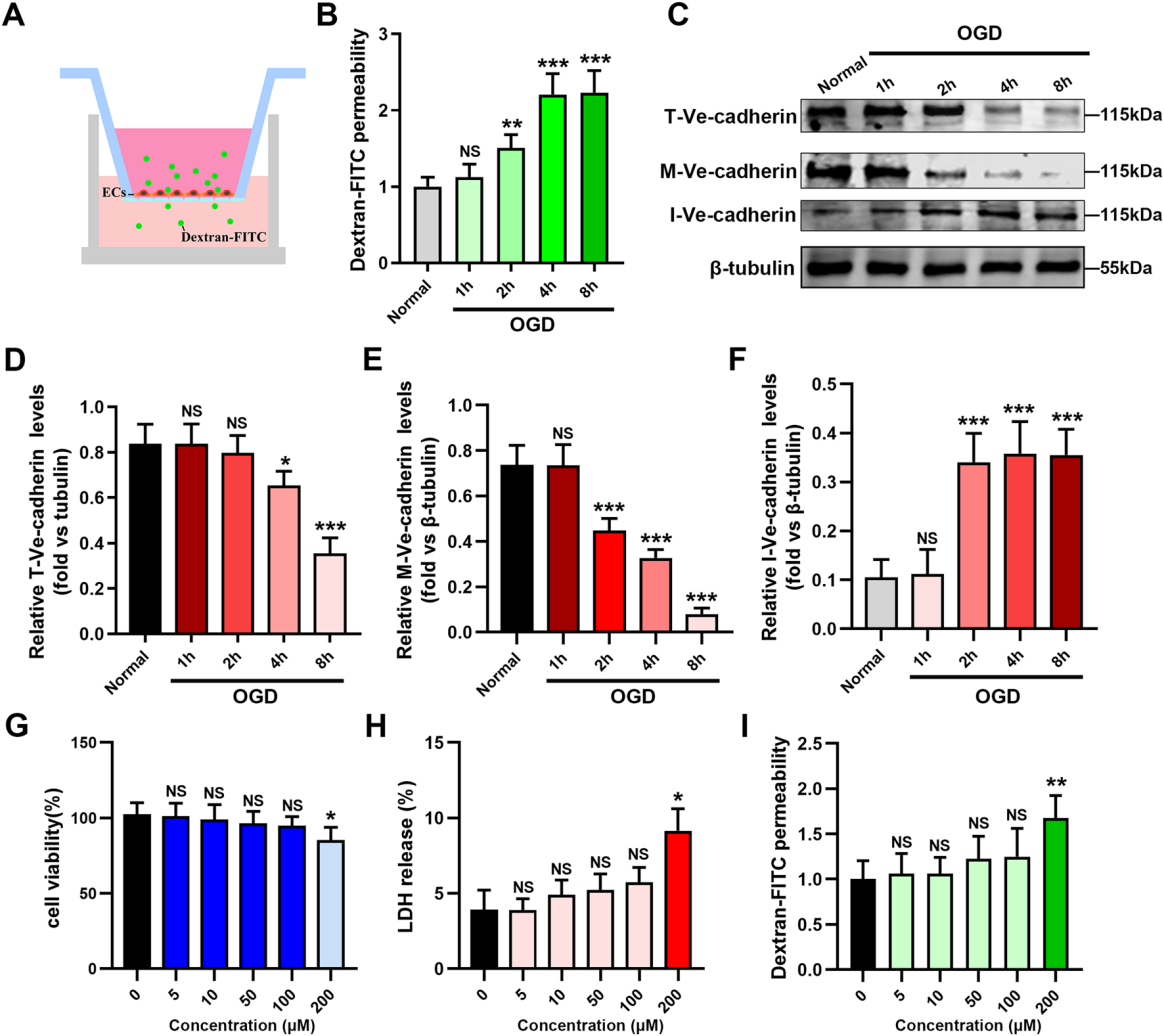
Establishment of an OGD model and safe therapeutic concentrations of nuciferine. (**A**, **B**) Transwell assay for permeability of the endothelial cells. n = 4. (**C**) Western blotting to detect the effect of OGD on the expression of Ve-cadherin in HCMECs. (**D**, **E**, **F**) Quantification of the related Ve-cadherin expression. T-Ve-cadherin, Ve-cadherin total protein; M-Ve-cadherin, Membrane Ve-cadherin; I-Ve-cadherin, Intracellular Ve-cadherin. n = 4. The effect of different concentrations of nuciferine on cell survival (**G**), apoptosis (**H**), and permeability (**I**) in HCMECs. n = 4. Data are expressed as the mean ± SD. NS, No significance (*P* > 0.05); *P < 0.05, **P < 0.01, ***P < 0.001 *vs.* Normal group.

Acute ischemia and hypoxia can destroy endothelial cells structurally and functionally, resulting in increased permeability. To further evaluate the effect of AMI injury on the endothelial barrier, we performed the transwell assay using HCMECs under the OGD condition (Fig. 3A). The permeability of the HCMECs changed significantly after OGD for 2 h (Fig. 3B).

Intercellular adhesion junctions (AJs) are a major determinant of the integrity of the endothelial barrier of the vascular bed^19^. The adhesion protein VE-cadherin, a structural protein characteristic of the intercellular adhesion junction structure, is considered the main signaling protein controlling the integrity of the endothelial barrier^20^. Decreased expression or intracellular translocation of this protein affects the endothelial barrier function^21^. To further assess the effect of nuciferine on AJs between vascular endothelial cells during AMI, we first detected the expression of VE-cadherin to determine the vascular junction structure. No significant change was noted in the total protein expression of VE-cadherin after OGD for 2 h. The total protein expression of VE-cadherin slightly decreased after OGD for 4 h (down about 22%) and then significantly decreased after OGD for 8 h (down about 58%). Interestingly, intracellular VE-cadherin expression was significantly higher than that in the normal group after OGD for 2 h (increased by about 2.2 times), and endothelial cell permeability also increased significantly. This phenomenon was also observed in the OGD-4 h group (Fig. 3C–F). To exclude the effect of changes in total protein expression on intracellular proteins, we considered OGD for 2 h as the main observation time point.

Further, we investigated the side effects of nuciferine on endothelial cells. Concentrations above 10 μM of nuciferine have been suggested to have negative effects on cardiomyocytes^14^. However, endothelial cells exhibited good tolerance to nuciferine. The nuciferine concentration below 100 μM did not significantly affect the proliferation, apoptosis, or permeability of HCMECs (Fig. 3G–I).

### Nuciferine maintains the stability of vascular endothelial AJs under OGD conditions

**Figure 4:**
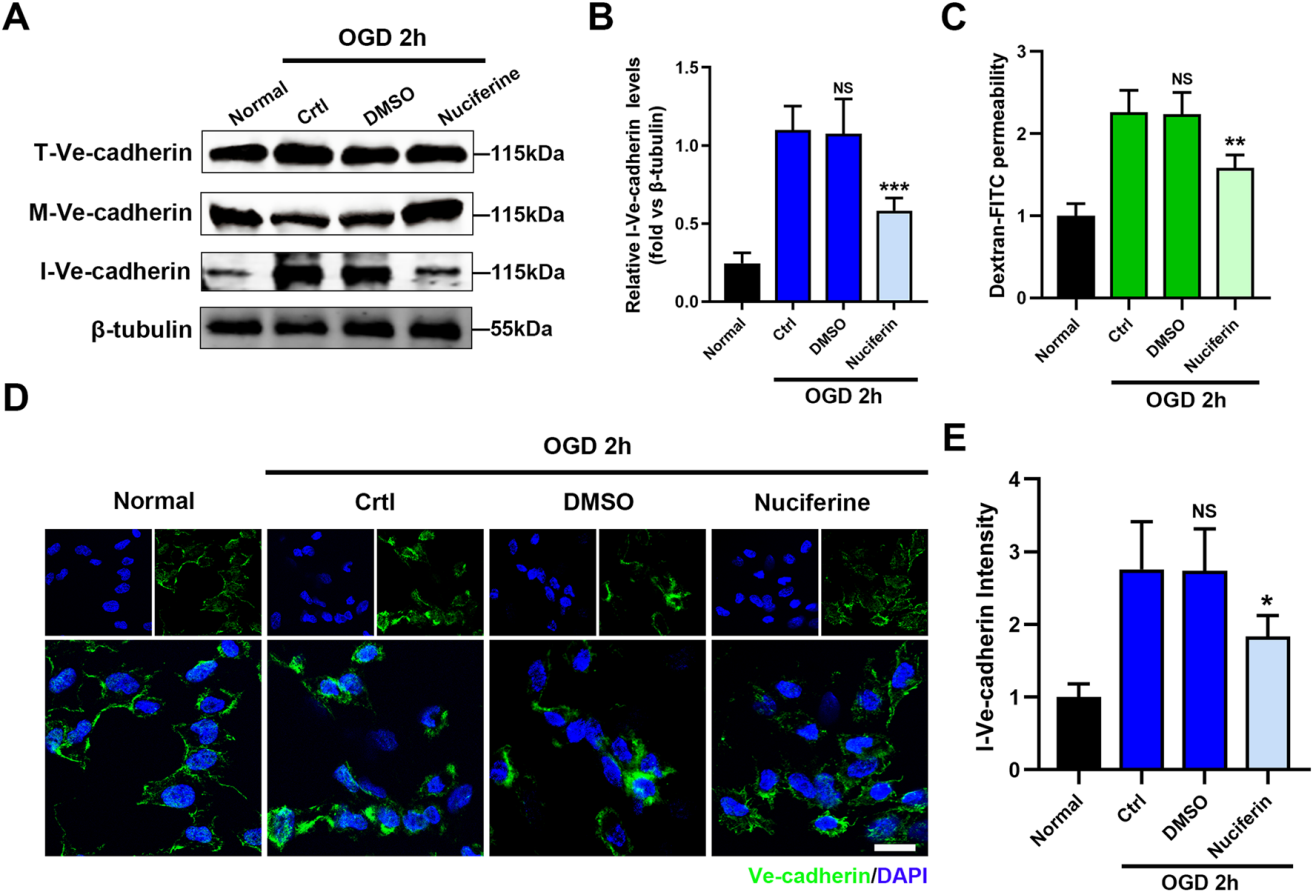
Nuciferine reduces leakage and maintains the stability of vascular endothelial AJs. (**A**, **B**) Western blotting was performed to detect the effect of nuciferine on the expression of intracellular Ve-cadherin in HCMECs. n = 4. (**C**) Transwell assay was performed to detect the permeability of HCMECs. n = 4. (**D**) Representative images of Ve-cadherin immunofluorescence staining of HCMECs under each of the indicated experimental conditions. n = 4. Scale bar = 10 μm. (**E**) Quantitative analysis of the intracellular Ve-cadherin fluorescence intensity. n = 4. Data are expressed as the mean ± SD. NS, No significance (*P* > 0.05); *P < 0.05, **P < 0.01, ***P < 0.001 *vs*. Ctrl group.

To investigate whether nuciferine exerts beneficial effects on endothelial cells, the effect of nuciferine on the intracellular translocation of VE-cadherin was first examined in HCMECs under OGD conditions. Nuciferine effectively inhibited the intracellular expression of VE-cadherin, reduced it by approximately 47% (Fig. 4A,B). Similarly, transwell results revealed that nuciferine effectively blocks an OGD-induced increase in endothelial cell permeability (Fig. 4C). Consistent with this result, the results of immunofluorescence staining demonstrated that the VE-cadherin protein in the normal group was neatly distributed, the intercellular connection was tight, and the cell structure was complete. Under OGD conditions, VE-cadherin distribution in the cell membrane was disrupted and distinct endocytosis occurred in the cytoplasm, which was effectively prevented by nuciferine (Fig. 4D,E). Nuciferine thus played a positive role in stabilizing endothelial cell AJs and reducing vascular permeability after MI in the mice.

### Nuciferine affects the distribution of VE-cadherin in endothelial cells through the PI3K/AKT pathway

**Figure 5:**
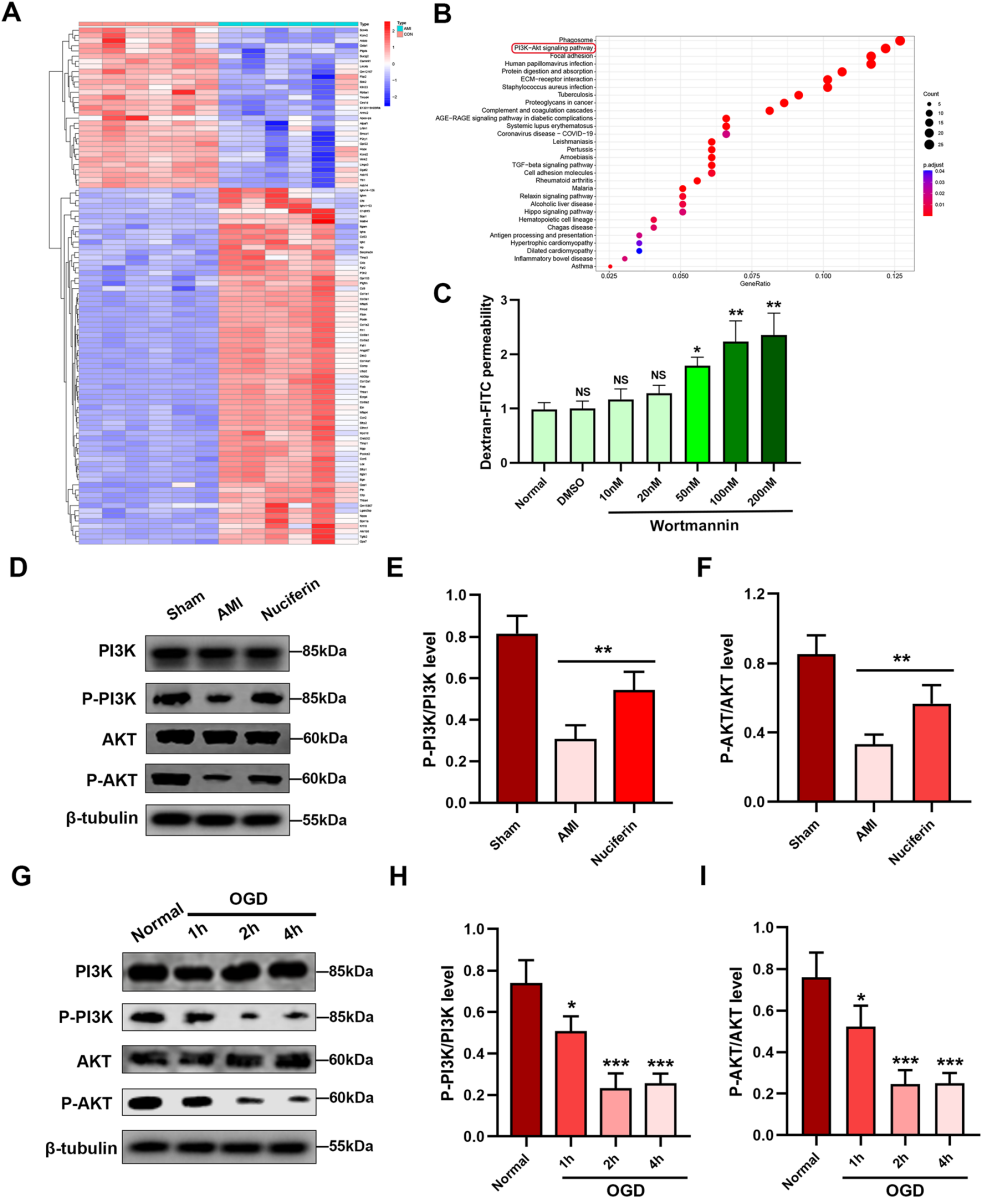
Nuciferine upregulated the phosphorylation levels of the PI3K/AKT pathway. (**A**) Heatmap of differential genes, data set GSE147365, including 6 control samples and 6 heart tissues from mice with acute myocardial infarction. (**B**) KEGG enrichment analysis of differential genes. (**C**) Transwell assay was performed to detect the effects of different concentrations of wortmannin (0, 10 nM, 20 nM, 50 nM, 100 nM, 200 nM) on HCMECs permeability. n = 3. (**D**) Western blotting was performed to detect the effect of nuciferine on the expression of related proteins in the myocardial tissues. (**E**, **F**) Quantification of the phospho-PI3K and phospho-AKT expression. n = 4. (**G**) Western blotting to detect the expression of related proteins under OGD conditions in HCMECs. (**H**, **I**) The quantification of the phospho-PI3K and phospho-AKT expression. n = 4. Data are expressed as the mean ± SD. *NS* No significance (*P* > 0.05); *P < 0.05, **P < 0.01, ***P < 0.001 *vs*. Normal or indicated group.

**Figure 6:**
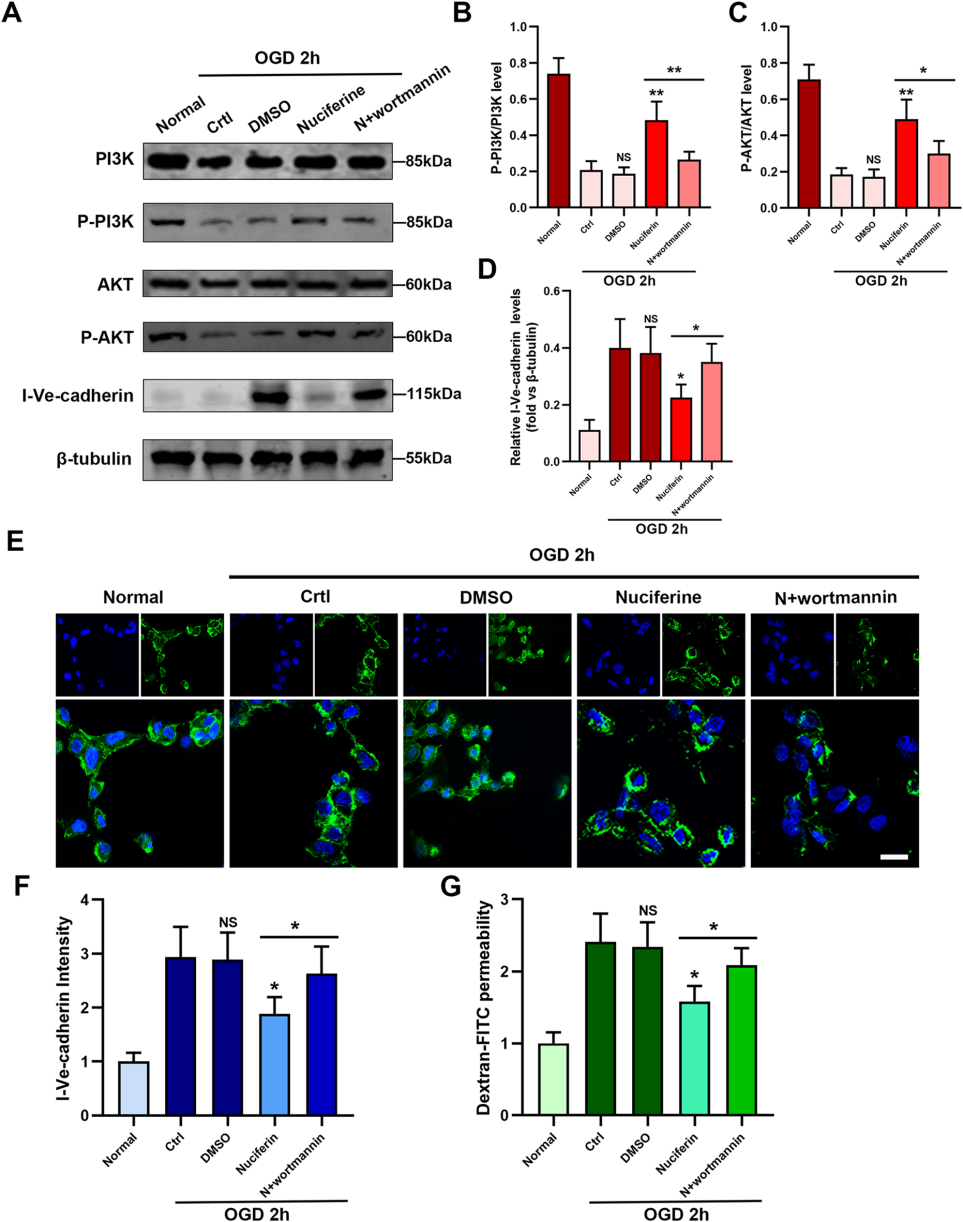
Nuciferine protects the endothelial barrier by regulating the PI3K/AKT pathway. (**A**) Western blotting was performed to detect the effect of nuciferine on the expression of related proteins (in the presence or absence of wortmannin) in HCMECs. (**B**, **C**, **D**) The quantification of the phospho-PI3K, phospho-AKT expression, and intracellular Ve-cadherin. n = 4. (**E**) Representative images of Ve-cadherin immunofluorescence staining of HCMECs under each of the indicated experimental conditions. Scale bar = 10 μm. (**F**) Quantitative analysis of the intracellular Ve-cadherin fluorescence intensity of HCMECs. n = 4. (**G**) The quantification of the permeability of HCMECs under each of the indicated experimental conditions. n = 4. Data are expressed as the mean ± SD. NS, No significance (*P* > 0.05); *P < 0.05, **P < 0.01 *vs*. Ctrl or indicated group.

Abnormal phosphorylation of PI3K/AKT pathway plays an important role in myocardial ischemic injury^22,23^. We reanalyzed the transcriptome information from the public database of normal and AMI mice (GSE147365 dataset), and the KEGG results showed high enrichment of differential genes in the PI3K/AKT signaling pathway (Fig. 5A,B). In addition, concentrations of wortmannin (an inhibitor of the PI3K/AKT pathway) exceeding 50 nM can notably enhance the permeability of endothelial cells (Fig. 5C). We also investigated the levels of the PI3K/AKT pathway protein in the AMI mice, the results showed that the level of PI3K/AKT pathway protein was significantly decreased in AMI mice, and a similar phenomenon was observed in endothelial cells under OGD conditions (Fig. 5D–F). We further investigated the significance of the PI3K/AKT pathway in endothelial cell permeability. The phosphorylation level of the PI3K/AKT pathway decreased in the OGD-1h group and remained low in the OGD-2h and OGD-4h groups (Fig. 5G–I). Nuciferine effectively increased the PI3K and AKT phosphorylation levels (Fig. 6A–D). This represents the positive regulatory effect of nuciferine on the PI3K/AKT pathway. The inhibitor wortmannin (50 nM) reversed the therapeutic effect of nuciferine, among them, nuciferine reduced the intracellular expression of VE-cadherin by 44%, but wortmannin increased it to 88% of the OGD 2 h-Ctrl group (Fig. 6A–G). These results suggest that the effect of nuciferine on the endothelial structure and function can be realized by activating the PI3K/AKT pathway.

## Discussion

**Figure 7:**
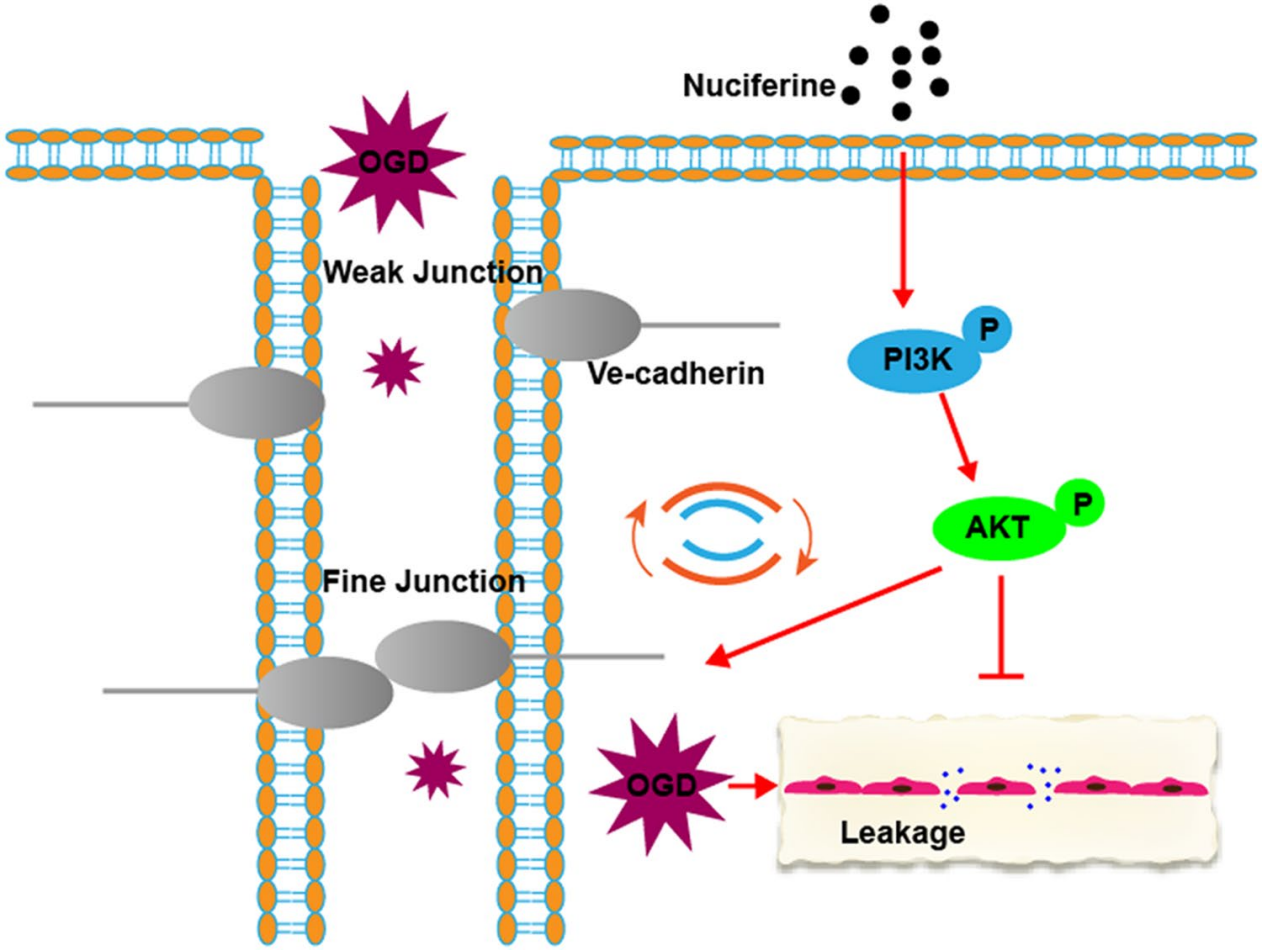
Schematic diagram demonstrating that nuciferine protects the endothelial barrier by regulating the PI3K/AKT pathway.

We investigated the protective effect of nuciferine on the microvascular structure after AMI and explored the potential underlying mechanism. Nuciferine can effectively maintain the microvascular structure and function after AMI, reduce vascular leakage, and alleviate myocardial damage, possibly by regulating the PI3K/AKT pathway (Fig. 7).

AMI can result in ischemia and hypoxia in various cardiac areas. The preserved perfusion function of the coronary microvascular network structure after hypoxia is closely related to the survival of myocardial cells, the occurrence of the no-reflow phenomenon, and the ventricular remodeling process, which directly determines the prognosis of AMI patients^24^. Hypoxia-induced endothelial AJ injury is the main pathological manifestation of AMI^25^. The adhesion protein VE-cadherin maintains the characteristic structure of the connection between endothelial cells and controls the endothelial barrier integrity^21^. Mounting evidence has highlighted that VE-cadherin destruction increases endothelial space and permeability, stimulates the inflammatory cascade, aggravates extensive damage to the myocardial tissue and endothelial cells, and severely affects the prognosis of AMI patients^26^. A fully functional microvascular structure can help the ischemic myocardium to rapidly recruit effective collateral blood flow to support the blood oxygen demand of the ischemic myocardium. Nuciferine is an aromatic ring alkaloid extracted from lotus leaves^27^. It has anti-inflammatory, antioxidant, and other therapeutic properties^12^. Nuciferine has been reported to reduce isoproterenol-induced myocardial cell damage by inhibiting the inflammatory response of the myocardium tissue in rats^13^. We established a mouse AMI model by blocking the left anterior descending branch of the heart. We further studied the preventive and treatment effect of nuciferine on AMI-induced microvascular structure and function damage and clarified its possible mechanism. In our study, nuciferine increased the membranous expression of VE-cadherin during AMI and reduced its intracellular migration, thereby reducing endothelial cell permeability. More importantly, a general decrease in the MI size and vascular leakage, and improved cardiac function were noted in the mice, providing further supporting evidence for the role of nuciferine in improving the ischemic hypoxic microenvironment and maintaining endothelial connectivity in AMI.

AMI involves the activation of various signaling pathways. Among these, the reduction of PI3K/Akt phosphorylation is a typical feature^23^. Activation or restoration of PI3K/Akt phosphorylation can effectively alleviate AMI or myocardial reperfusion injury^28^. Nuciferine significantly increased PI3K/Akt phosphorylation in AMI. The corresponding inhibitor wortmannin blocked the therapeutic effect of nuciferine. These results suggest that PI3K/AKT pathway activation may be a crucial mechanism underlying endothelial barrier integrity and function during the protective effect exerted by nuciferine against AMI. Nuciferine is delivered to mice intragastrically before AMI occurrence. The intragastric route represents a convenient route of administration for potential myocardial protection and improved functional recovery.

Nuciferine may be more appropriate for "planned" ischemic events, such as after coronary artery bypass surgery or during chronic myocardial ischemic injury. Furthermore, our results revealed that the negative effects of nuciferine on endothelial cells are very small. Nuciferine has been shown to protect cardiomyocytes, but large doses of this drug may exert negative effects. Therefore, further research is required to determine a safe dose of nuciferine. We did not investigate the effects of nuciferine on female AMI mice because estrogen has been reported to have a potential protective effect on ischemic myocardium and microvascular structure^29^. Maintaining the stability of VE-cadherin between endothelial cells is the key to protecting the endothelial barrier and thus alleviating MI. Nuciferine alleviates myocardial cell damage and microvascular structural damage in mice. Although other mechanisms may be involved, our findings can potentially be used for the treatment of acute coronary syndromes.

## Conclusion

Our results indicated that nuciferine can be used as a drug protecting the microvascular structure and function after MI. Applying nuciferine during AMI is a feasible strategy for reducing vascular leakage and MI and improving cardiac function. Our findings may offer promising new ways to improve the management and prognostic strategies for AMI (Supplementary information).

### Supplementary Information

Supplementary Information 1.

Supplementary Information 2.

Supplementary Information 3.

## Data Availability

The datasets used and/or analyzed during the current study are available from the corresponding authors on reasonable request.
