# Supplementary figures and images for: Nuciferine reduces vascular leakage and improves cardiac function in acute myocardial infarction by regulating the PI3K/AKT pathway
Source: Sci Rep. 2024 Mar 26;14:7086. doi: 10.1038/s41598-024-57595-w (PMC10963720; doi:10.1038/s41598-024-57595-w)

**Figure3 C**

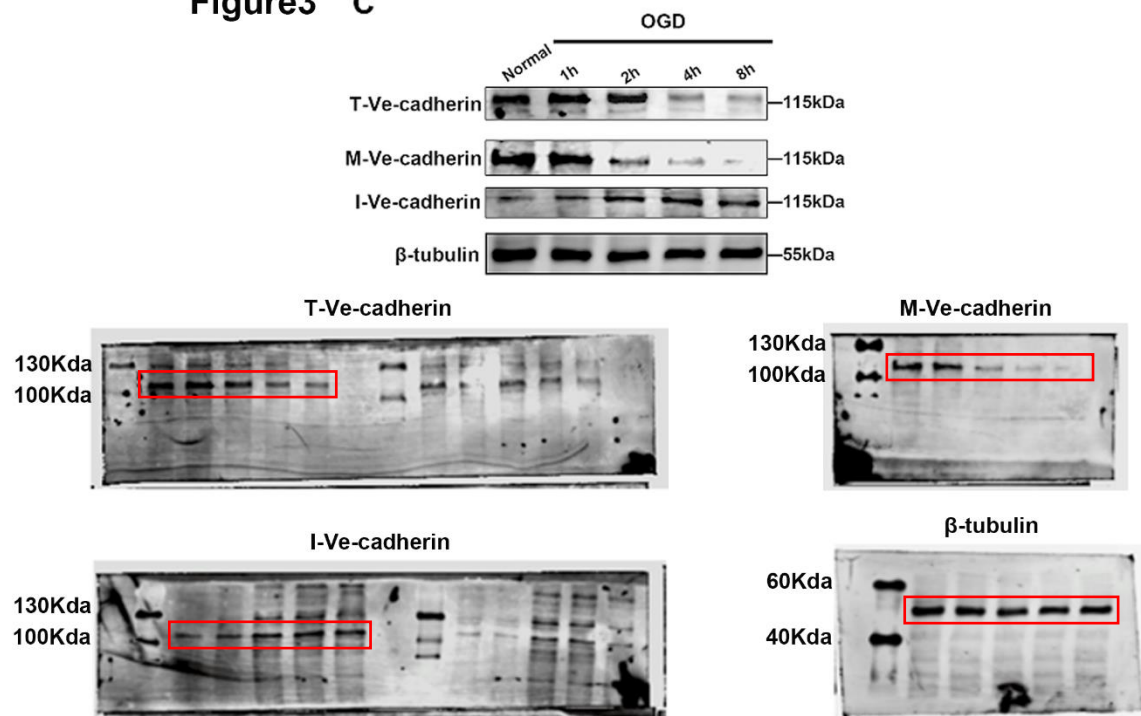

**Figure4 A**

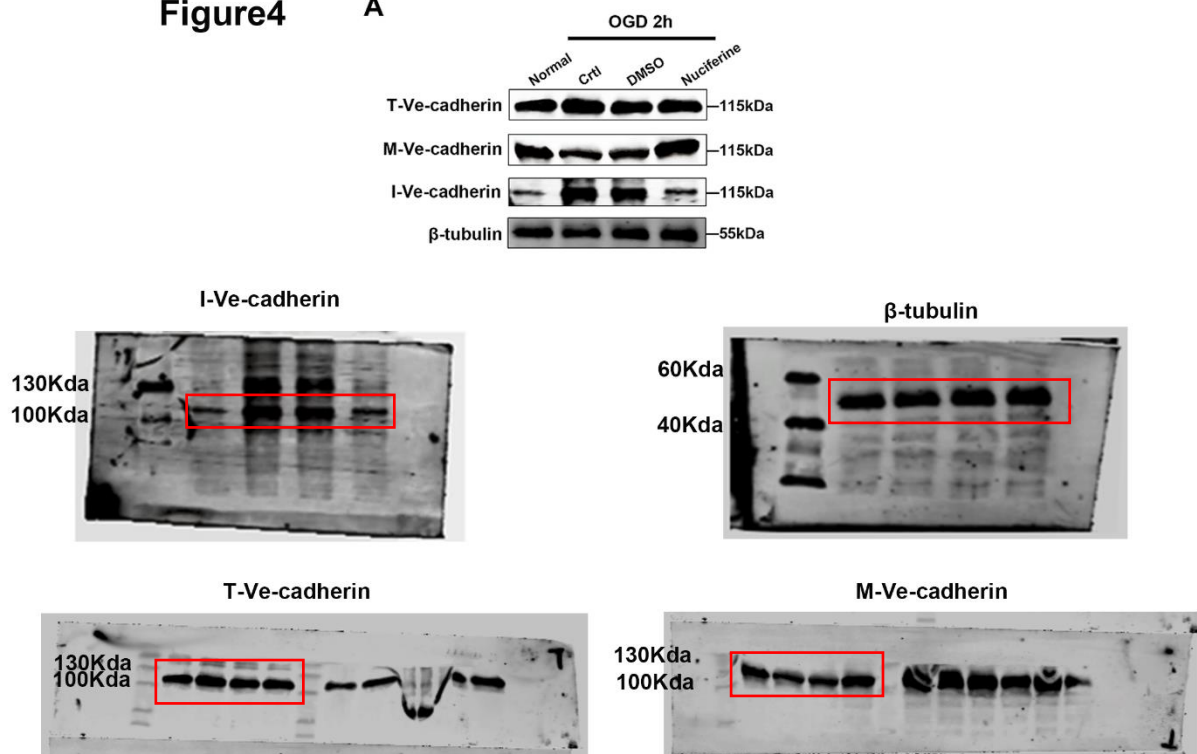

Figure5 D

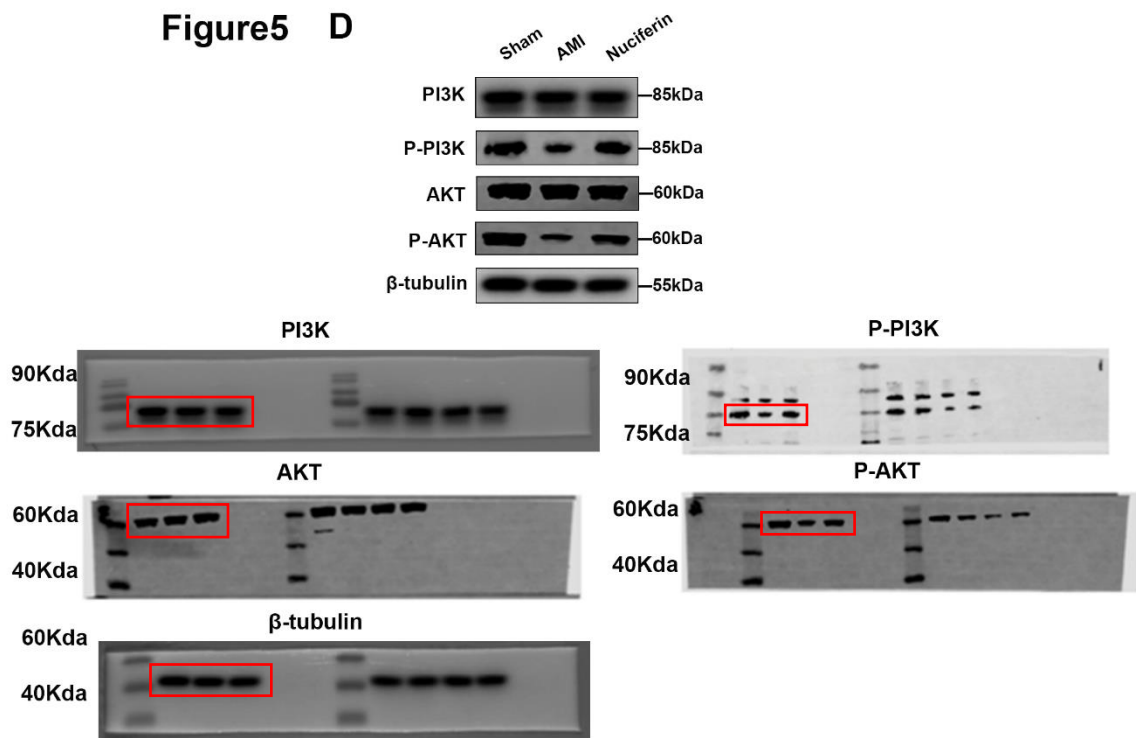

Figure5 G

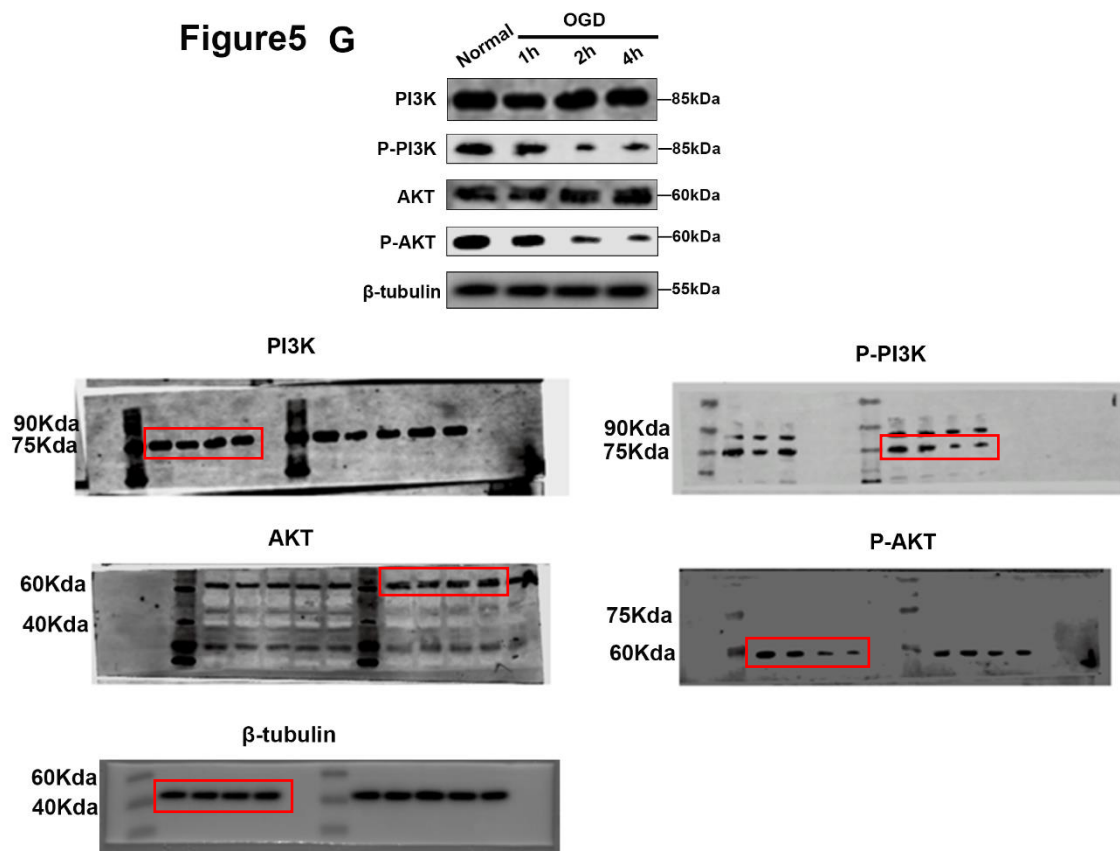

Figure6 A

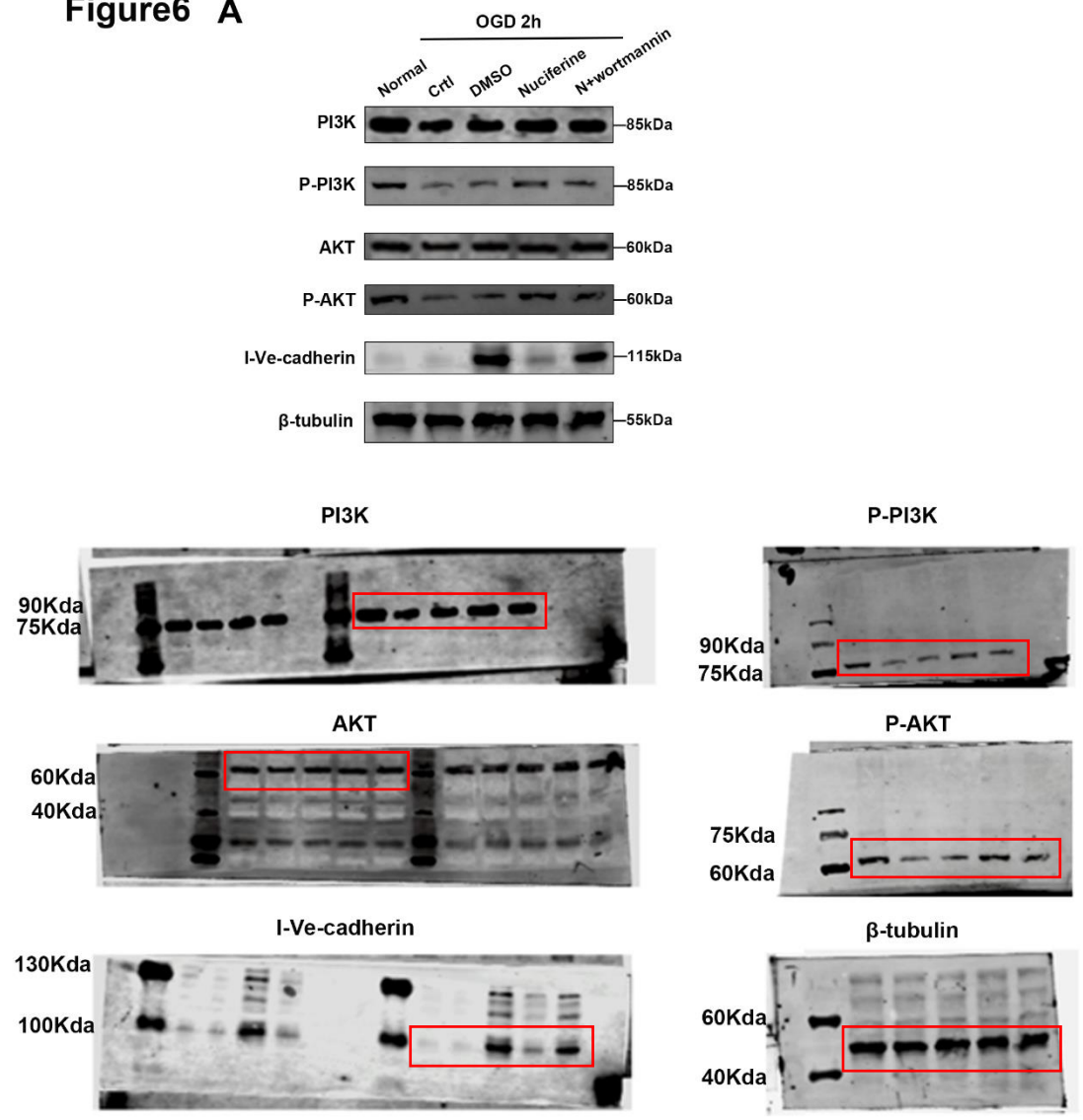

Supplement: Supplementary file 2 — Supplementary Information 2. [file 41598_2024_57595_MOESM2_ESM.pdf]

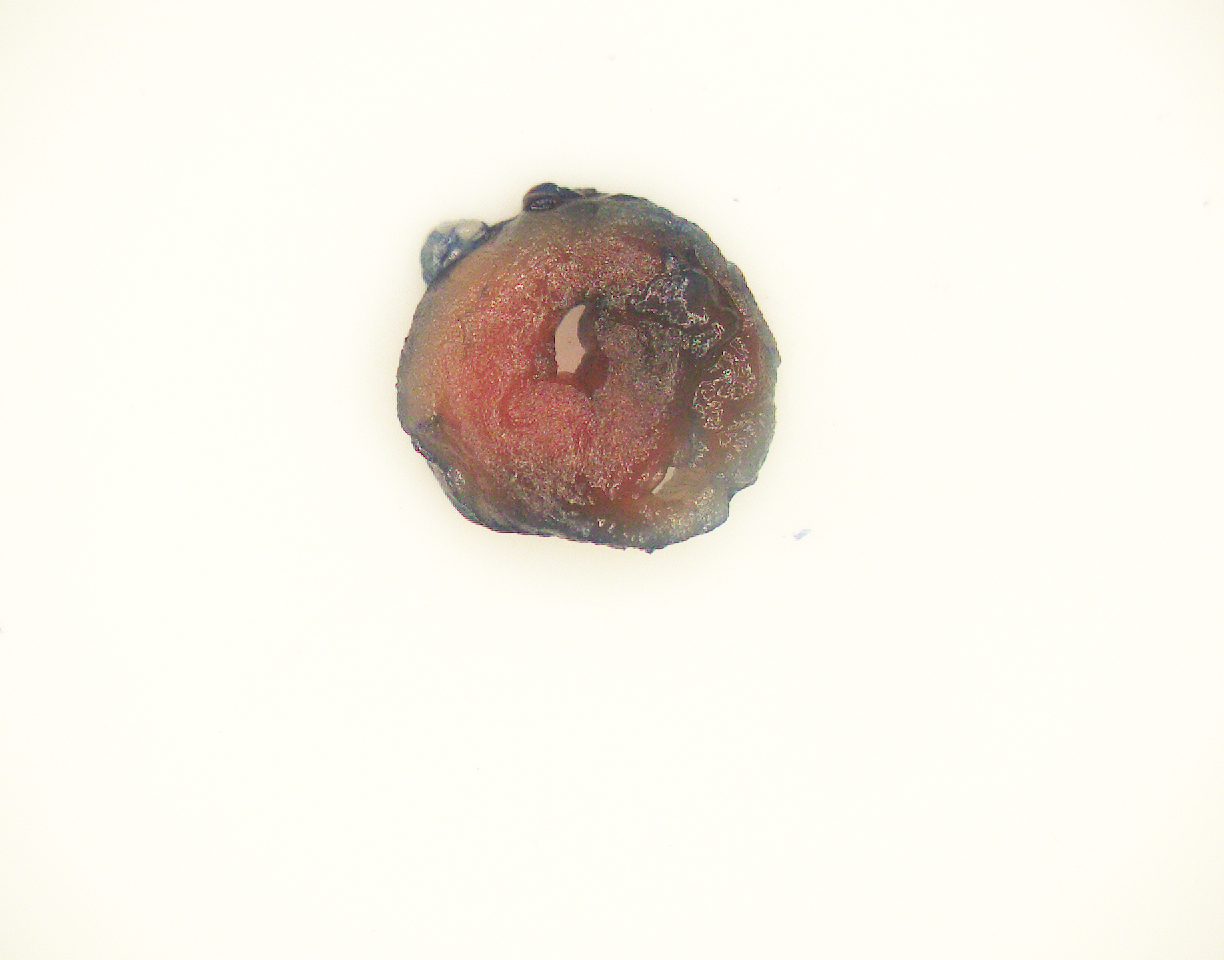

Supplement: Supplementary file 3 — Supplementary Information 3. [file 41598_2024_57595_MOESM3_ESM.zip › Raw figure/Figure2A/AMI.tif]

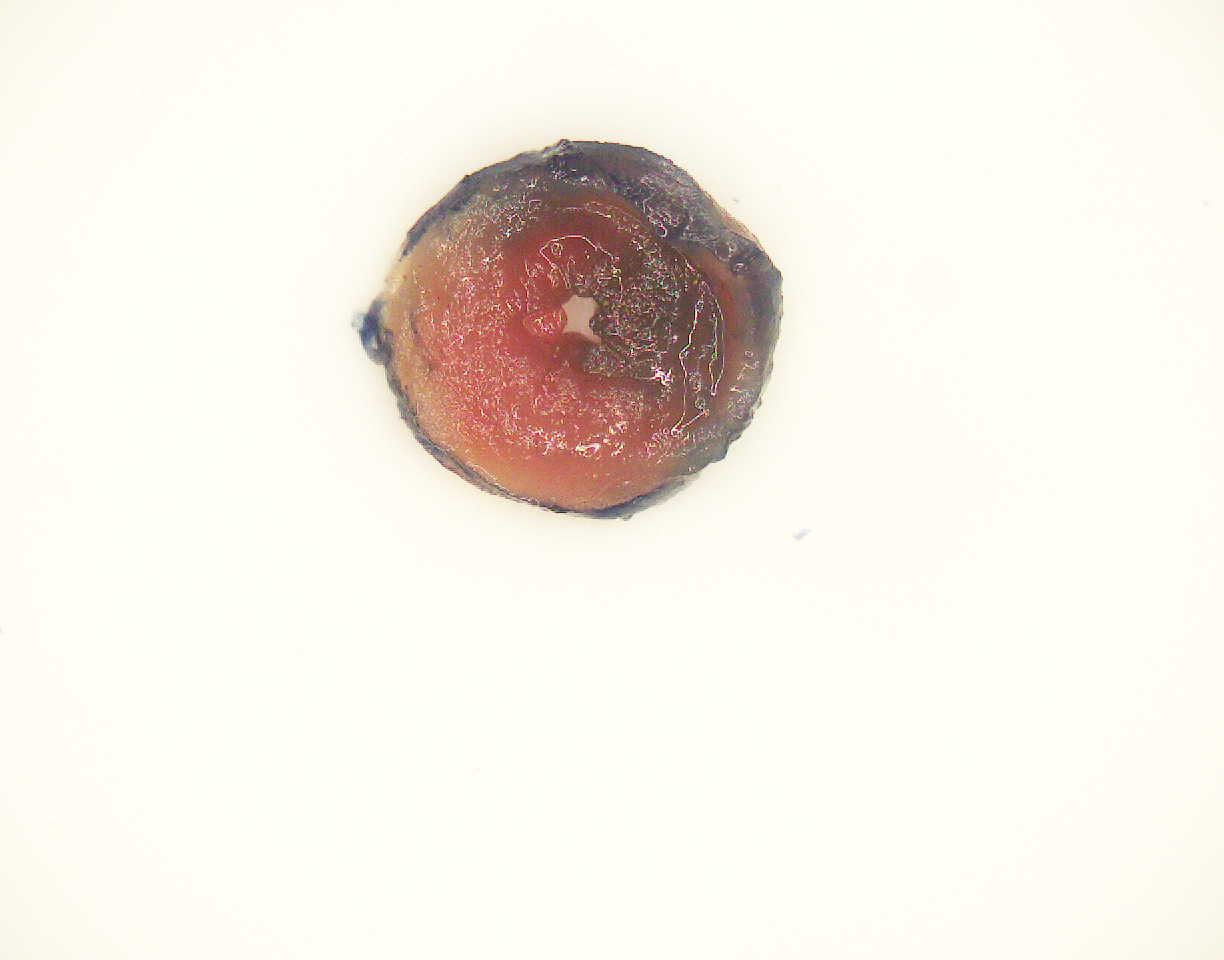

Supplement: Supplementary file 3 — Supplementary Information 3. [file 41598_2024_57595_MOESM3_ESM.zip › Raw figure/Figure2A/AMI+Nuciferine.tif]

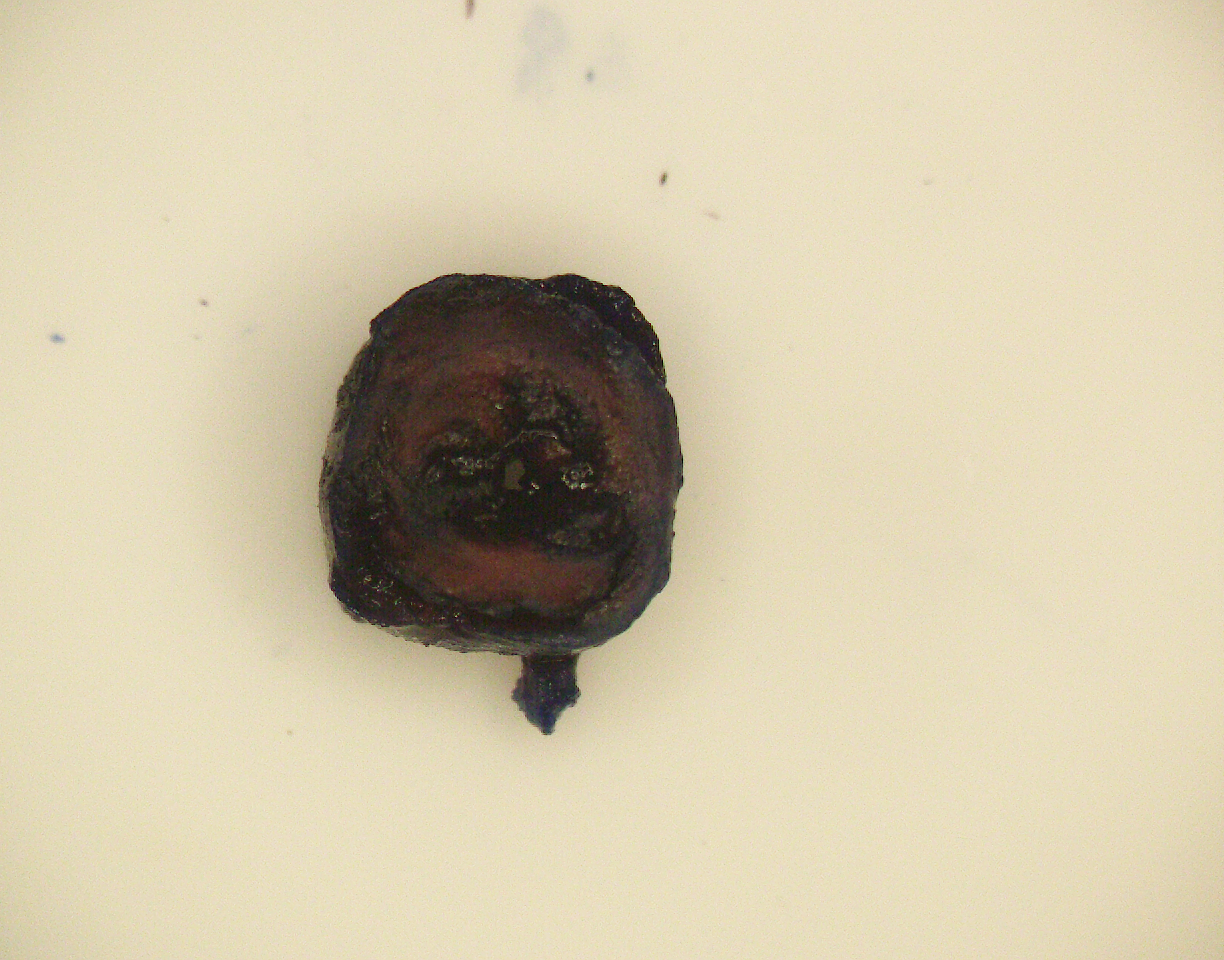

Supplement: Supplementary file 3 — Supplementary Information 3. [file 41598_2024_57595_MOESM3_ESM.zip › Raw figure/Figure2A/Nuciferine.tif]

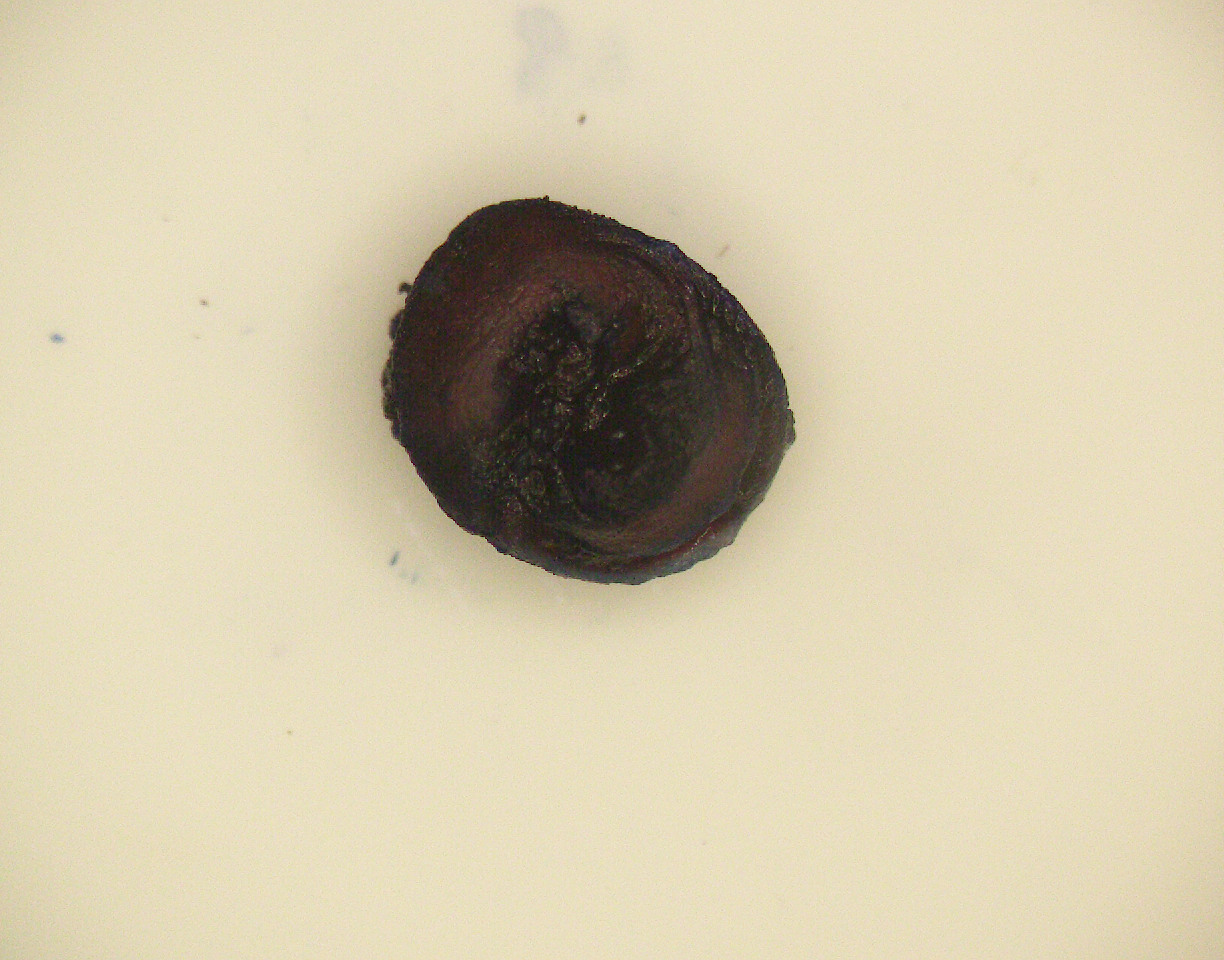

Supplement: Supplementary file 3 — Supplementary Information 3. [file 41598_2024_57595_MOESM3_ESM.zip › Raw figure/Figure2A/Sham.tif]

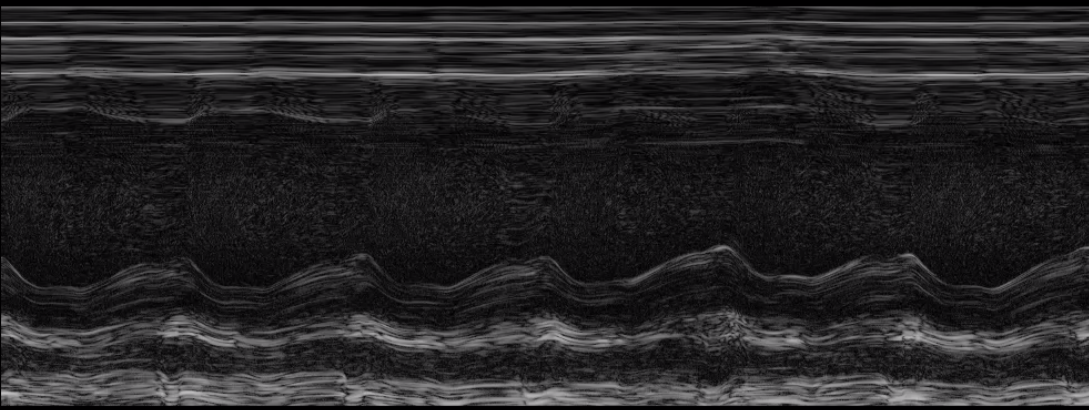

Supplement: Supplementary file 3 — Supplementary Information 3. [file 41598_2024_57595_MOESM3_ESM.zip › Raw figure/Figure2C/AMI.png]

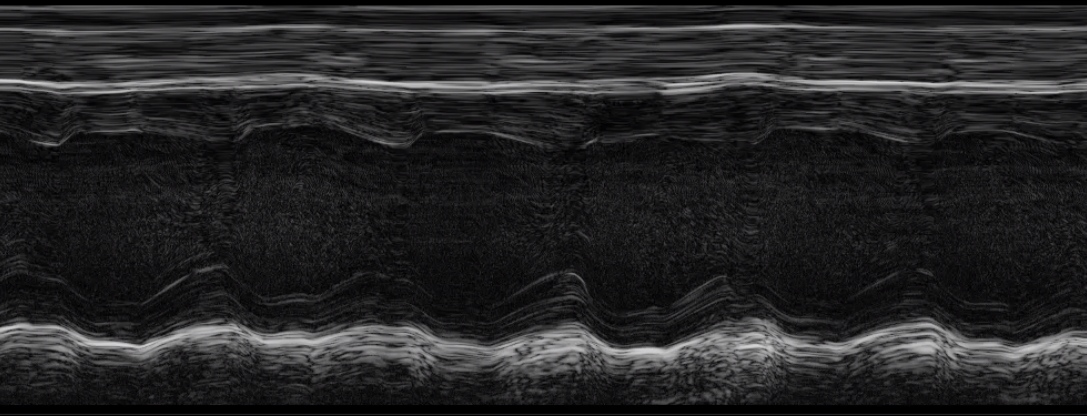

Supplement: Supplementary file 3 — Supplementary Information 3. [file 41598_2024_57595_MOESM3_ESM.zip › Raw figure/Figure2C/AMI+Nuciferine.png]

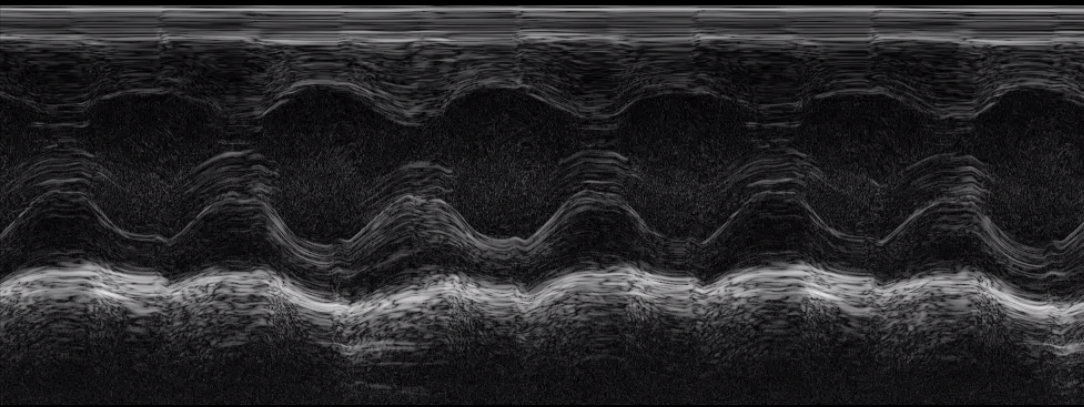

Supplement: Supplementary file 3 — Supplementary Information 3. [file 41598_2024_57595_MOESM3_ESM.zip › Raw figure/Figure2C/Nuciferine.png]

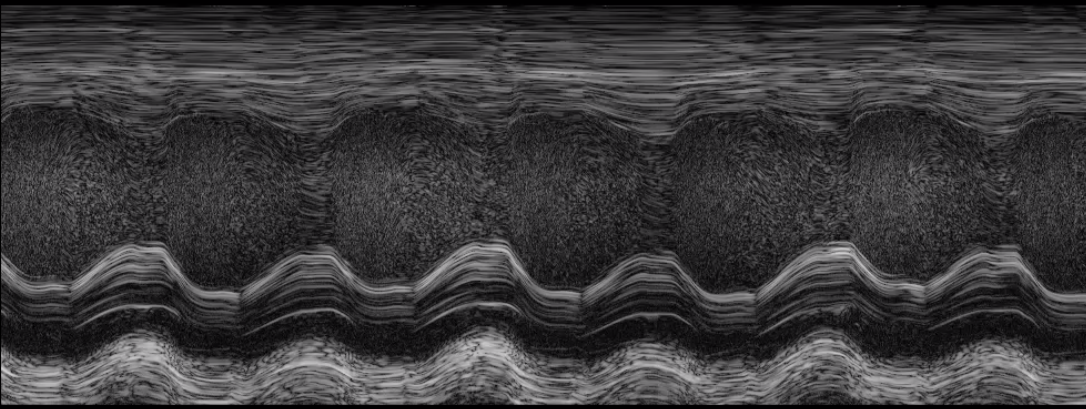

Supplement: Supplementary file 3 — Supplementary Information 3. [file 41598_2024_57595_MOESM3_ESM.zip › Raw figure/Figure2C/Sham.png]

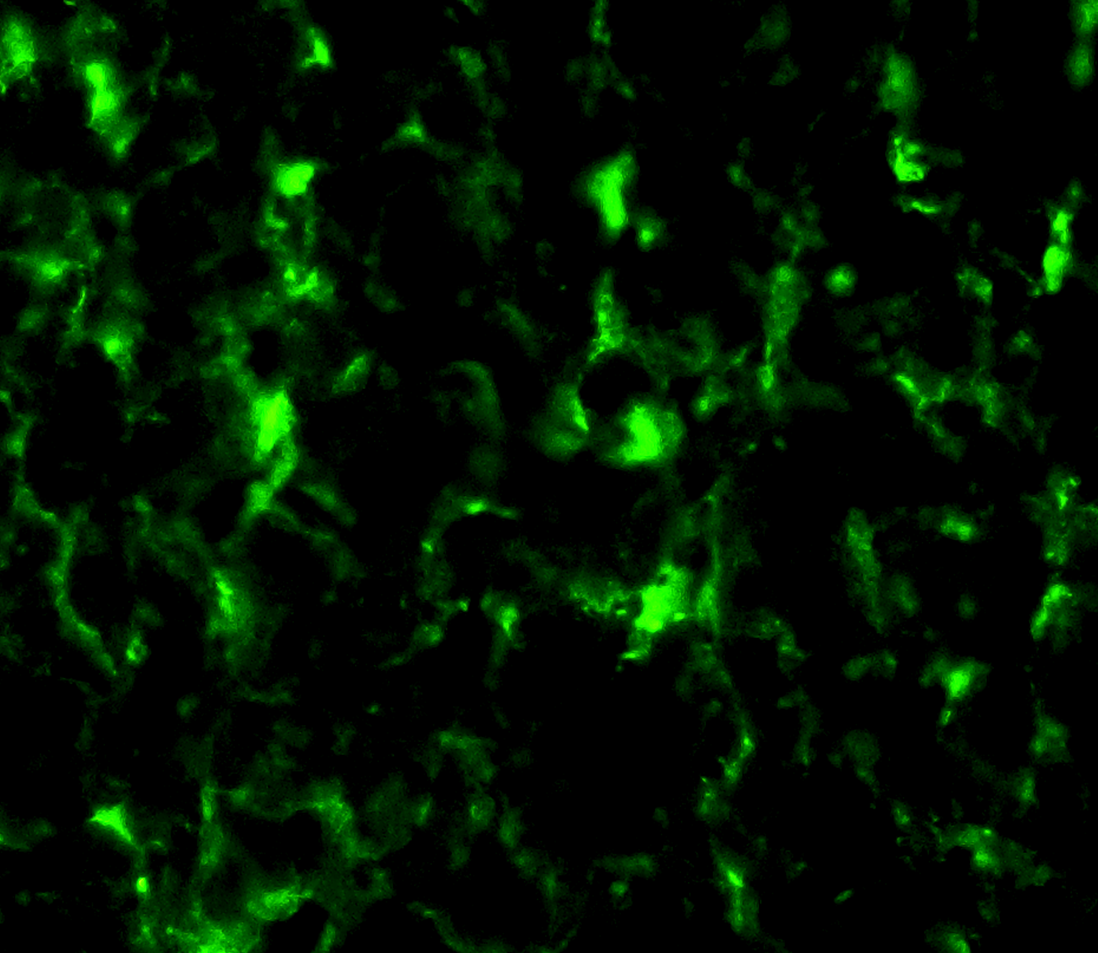

Supplement: Supplementary file 3 — Supplementary Information 3. [file 41598_2024_57595_MOESM3_ESM.zip › Raw figure/Figure2E/AMI/cd31.tif]

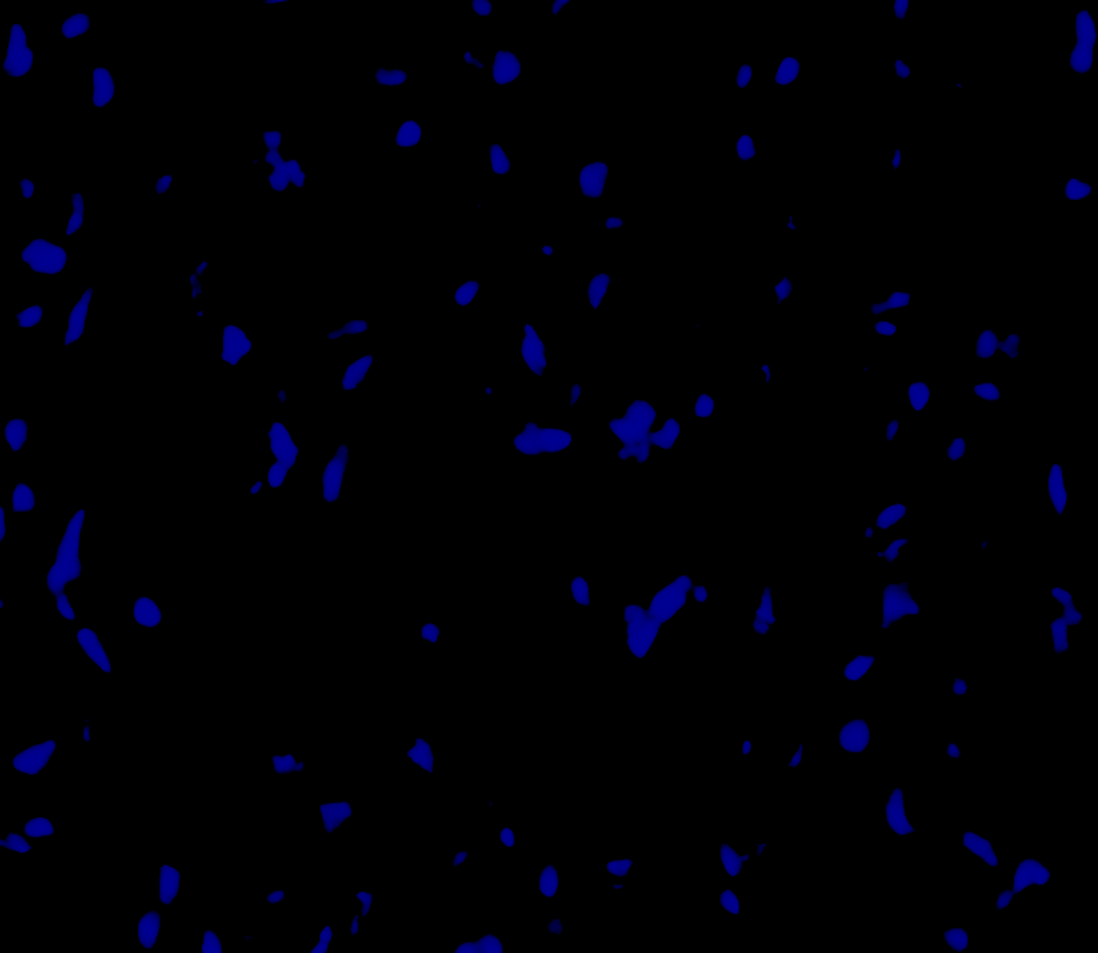

Supplement: Supplementary file 3 — Supplementary Information 3. [file 41598_2024_57595_MOESM3_ESM.zip › Raw figure/Figure2E/AMI/dapi.tif]

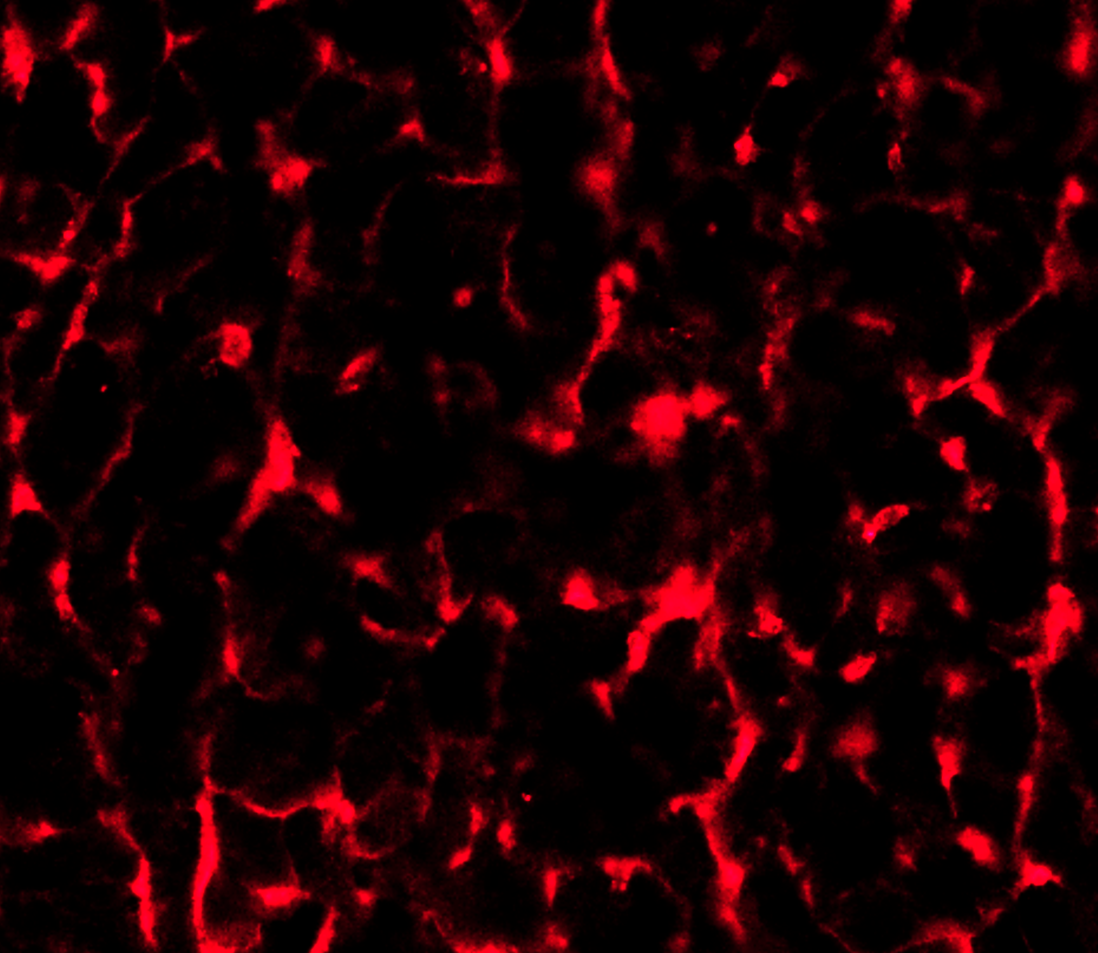

Supplement: Supplementary file 3 — Supplementary Information 3. [file 41598_2024_57595_MOESM3_ESM.zip › Raw figure/Figure2E/AMI/fn.tif]

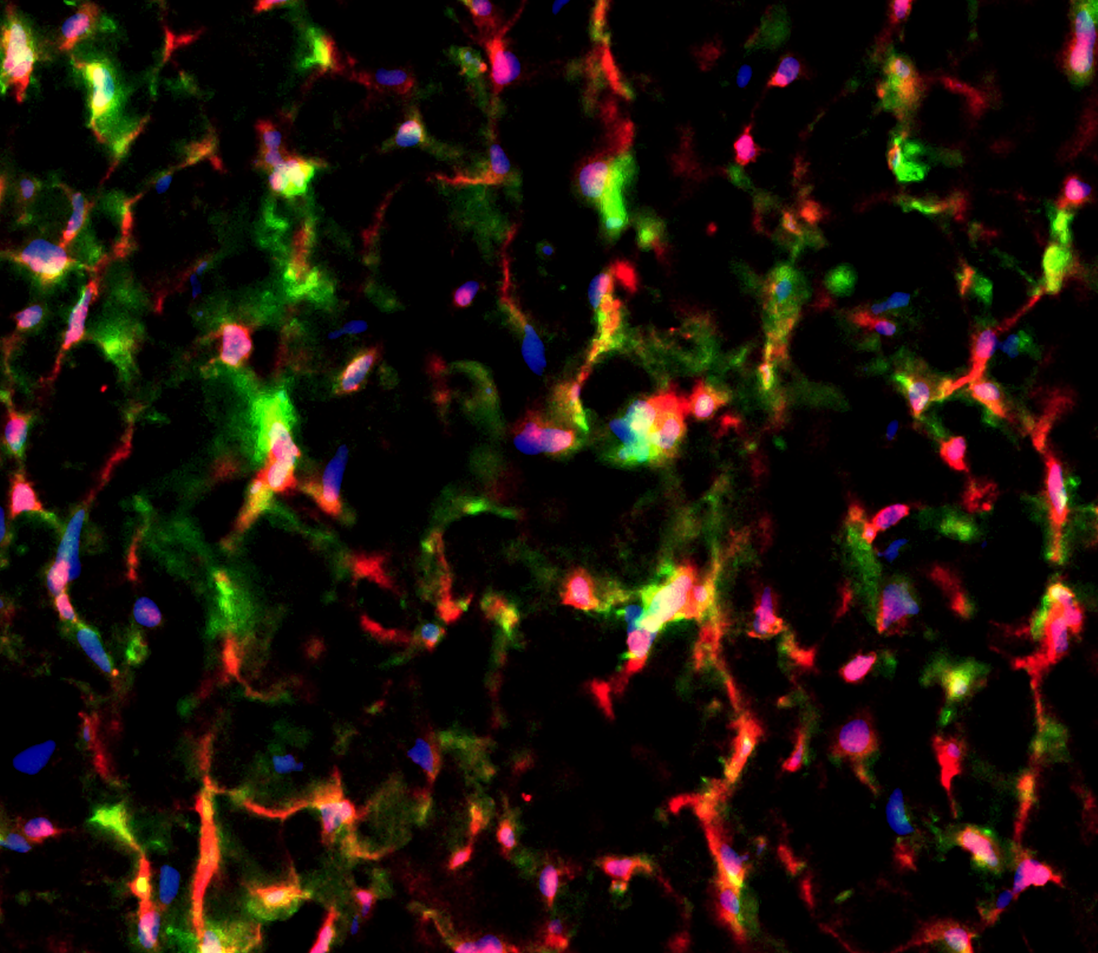

Supplement: Supplementary file 3 — Supplementary Information 3. [file 41598_2024_57595_MOESM3_ESM.zip › Raw figure/Figure2E/AMI/╬┤▒Ω╠Γ-3.tif]

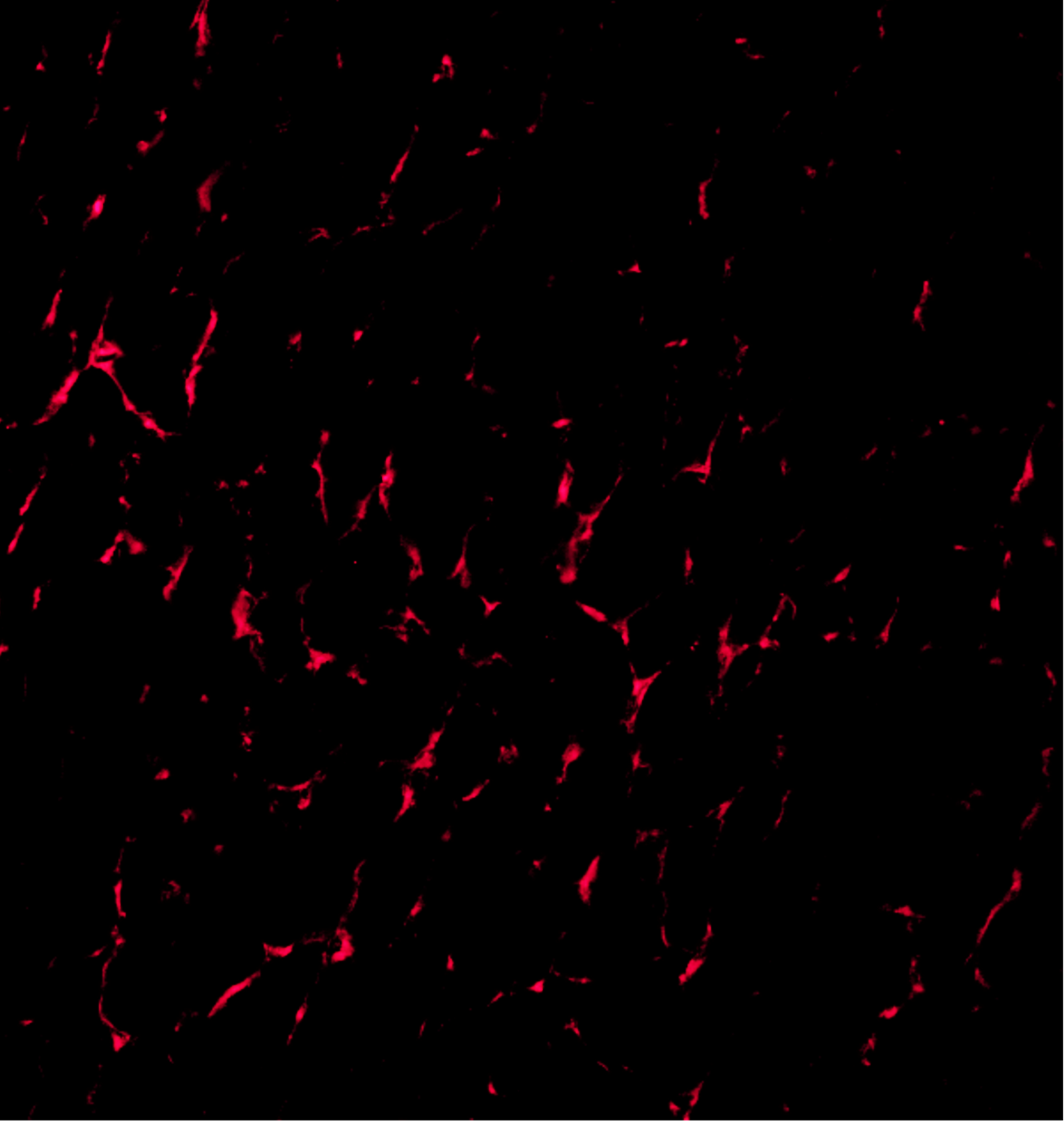

Supplement: Supplementary file 3 — Supplementary Information 3. [file 41598_2024_57595_MOESM3_ESM.zip › Raw figure/Figure2E/AMI+Nuciferine/152.tif]

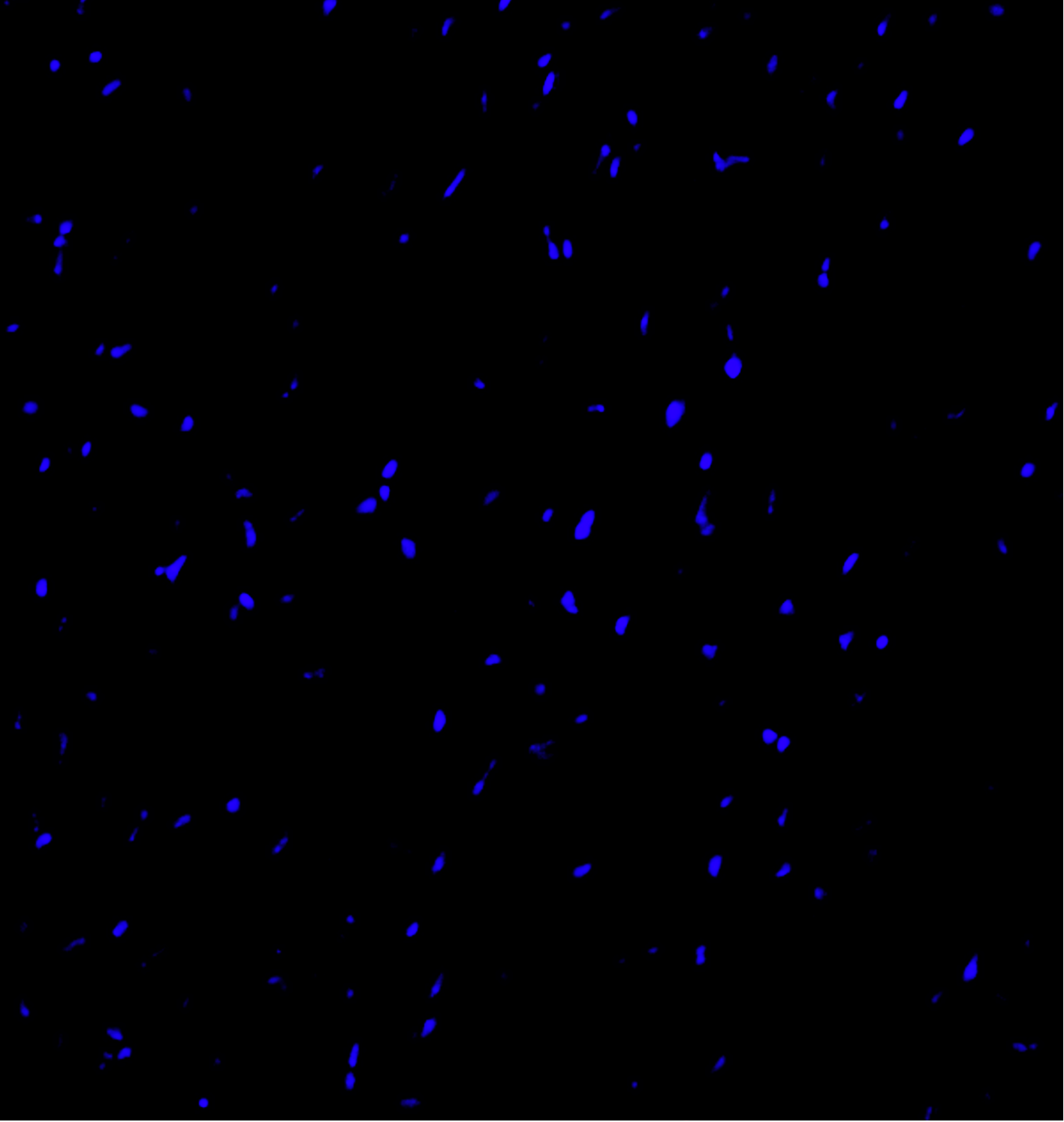

Supplement: Supplementary file 3 — Supplementary Information 3. [file 41598_2024_57595_MOESM3_ESM.zip › Raw figure/Figure2E/AMI+Nuciferine/45.tif]

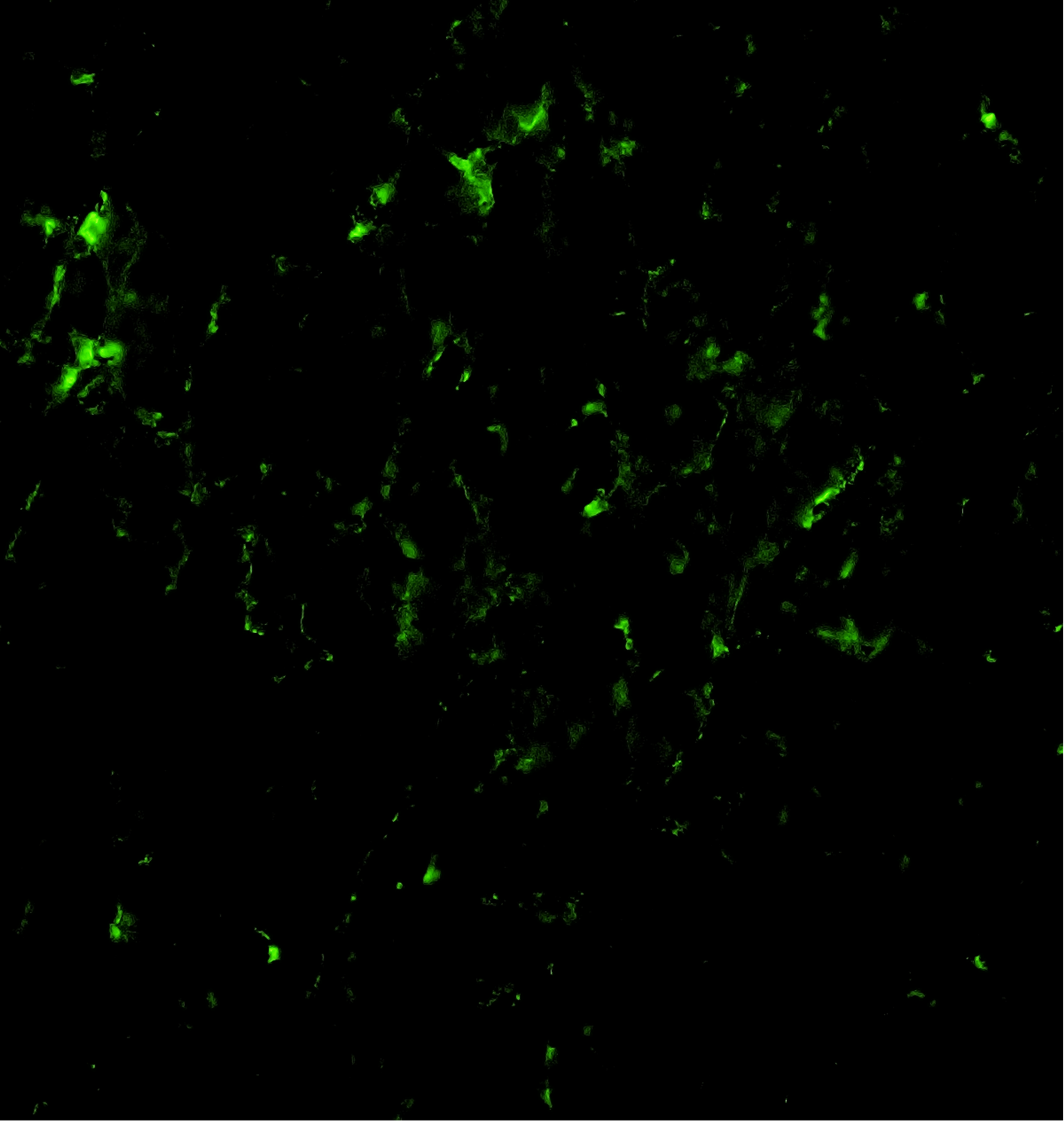

Supplement: Supplementary file 3 — Supplementary Information 3. [file 41598_2024_57595_MOESM3_ESM.zip › Raw figure/Figure2E/AMI+Nuciferine/54.tif]

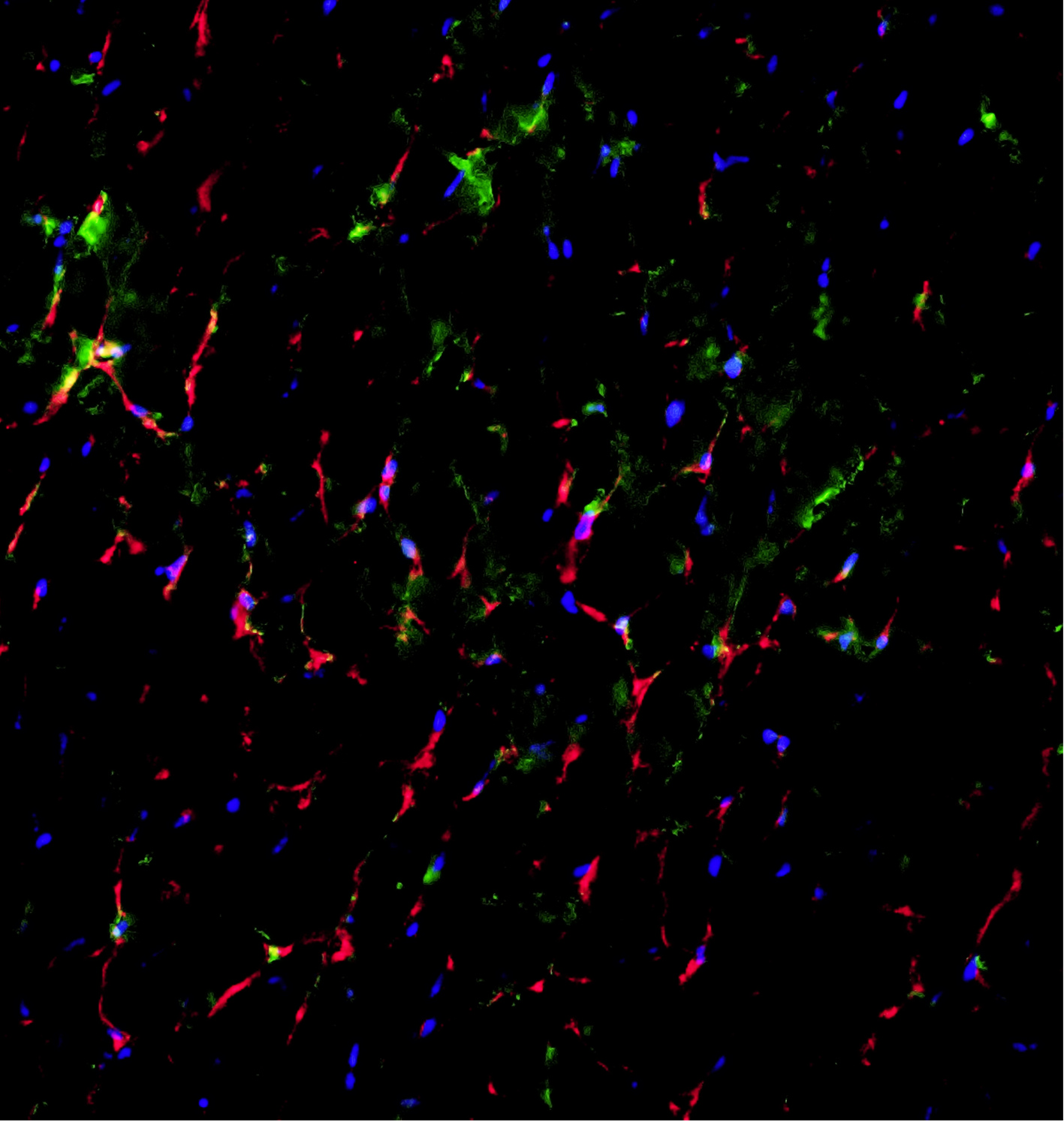

Supplement: Supplementary file 3 — Supplementary Information 3. [file 41598_2024_57595_MOESM3_ESM.zip › Raw figure/Figure2E/AMI+Nuciferine/645.tif]

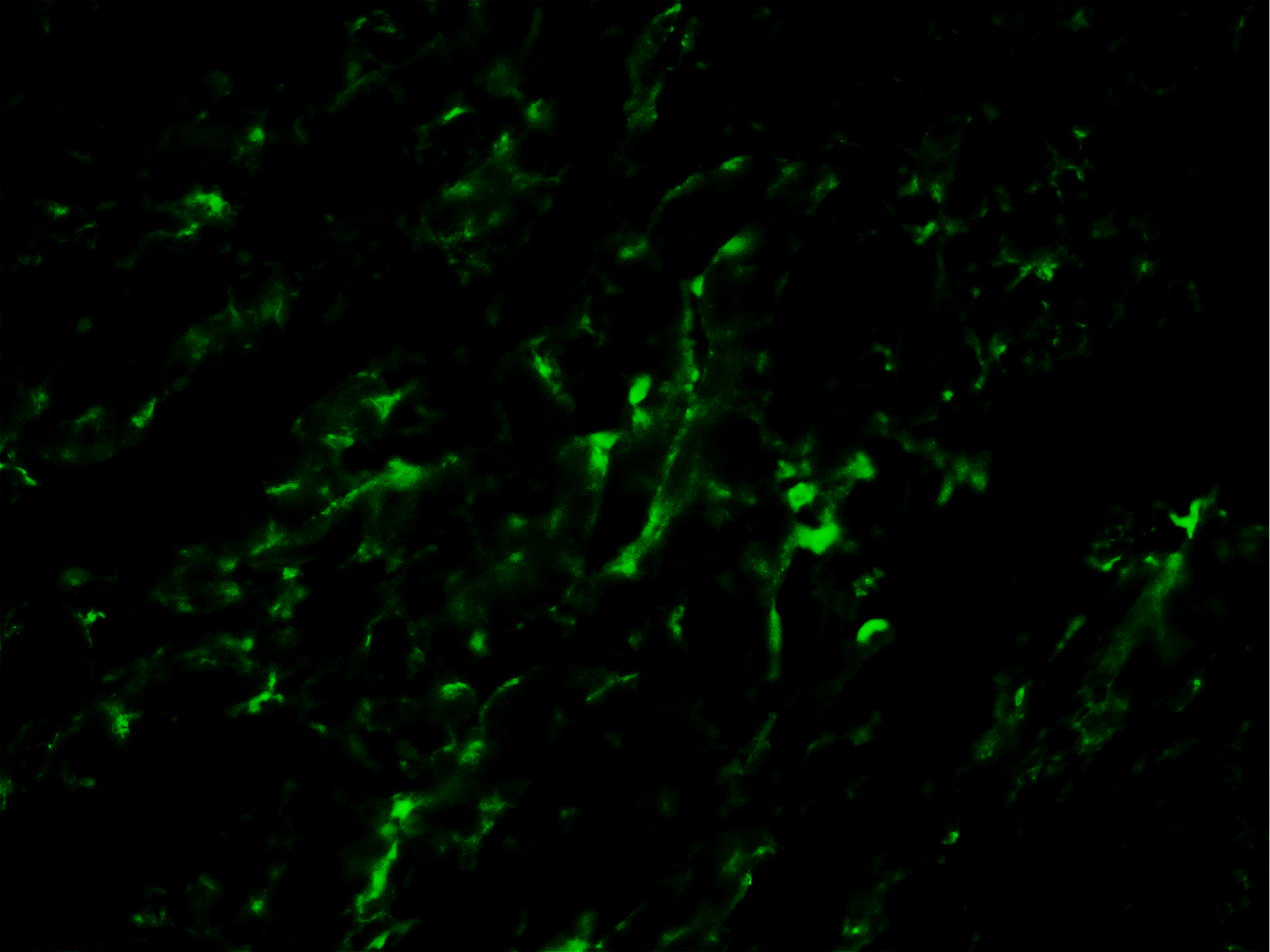

Supplement: Supplementary file 3 — Supplementary Information 3. [file 41598_2024_57595_MOESM3_ESM.zip › Raw figure/Figure2E/Nuciferine/cd31.tif]

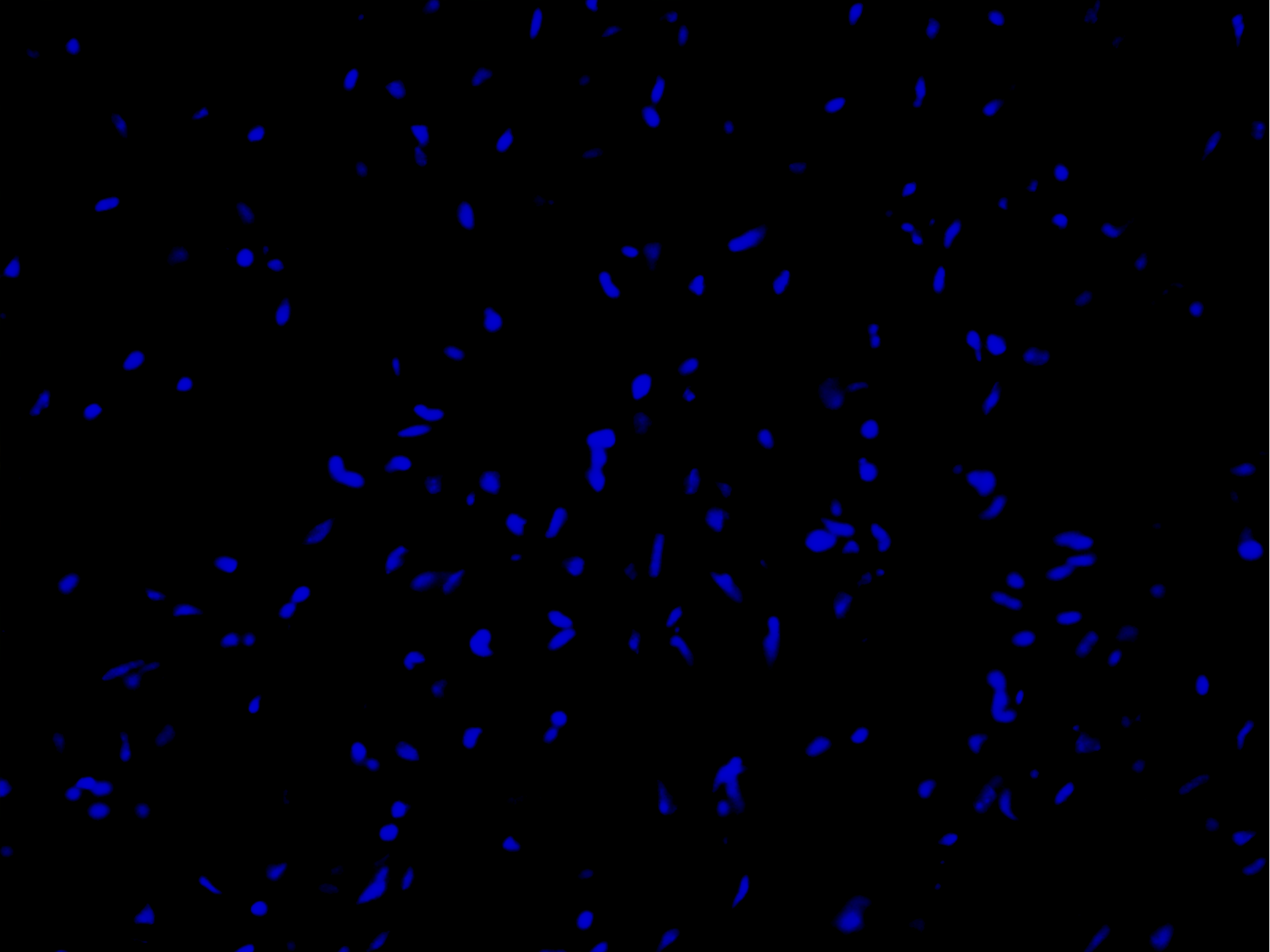

Supplement: Supplementary file 3 — Supplementary Information 3. [file 41598_2024_57595_MOESM3_ESM.zip › Raw figure/Figure2E/Nuciferine/dapi.tif]

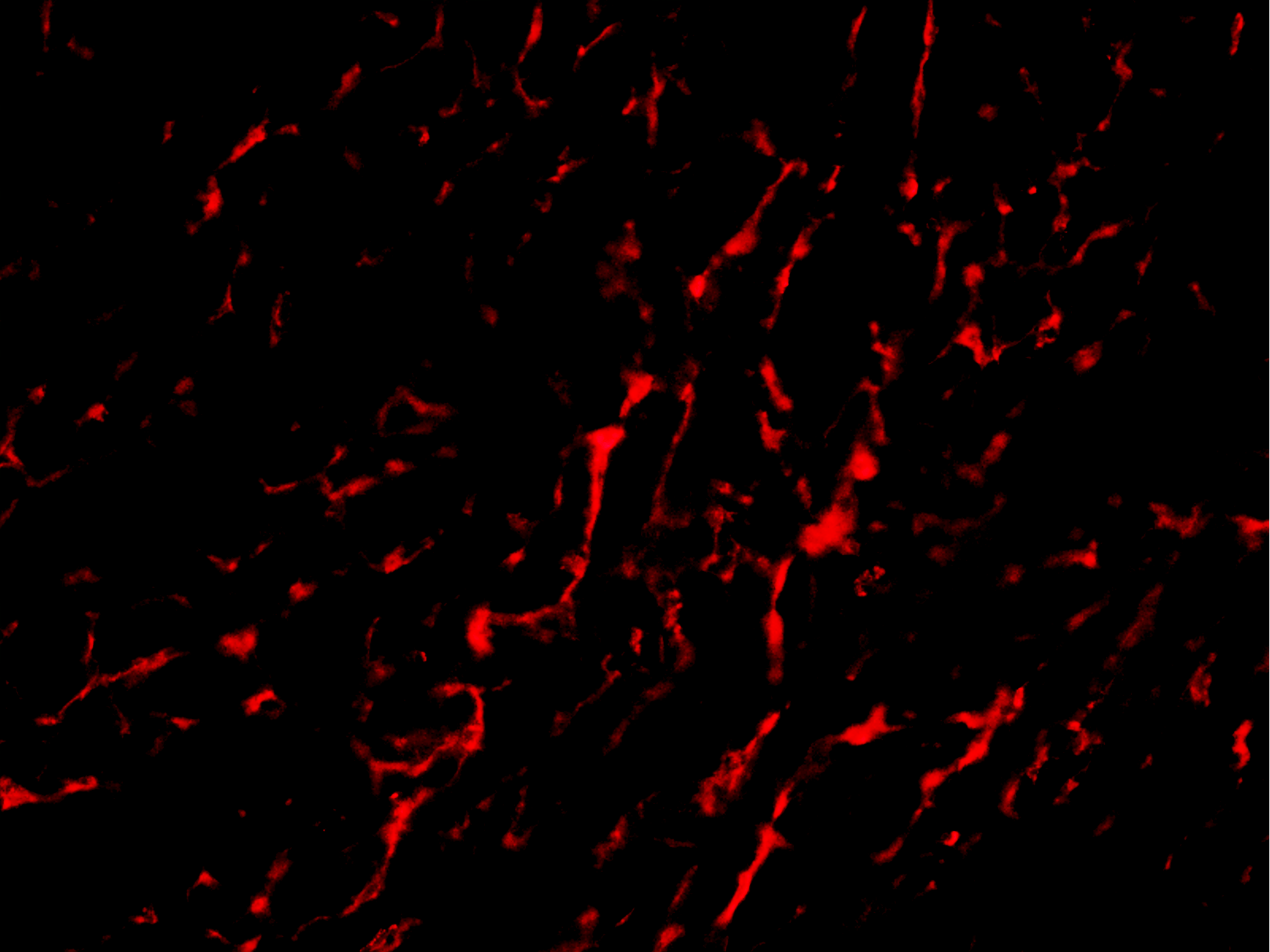

Supplement: Supplementary file 3 — Supplementary Information 3. [file 41598_2024_57595_MOESM3_ESM.zip › Raw figure/Figure2E/Nuciferine/fn.tif]

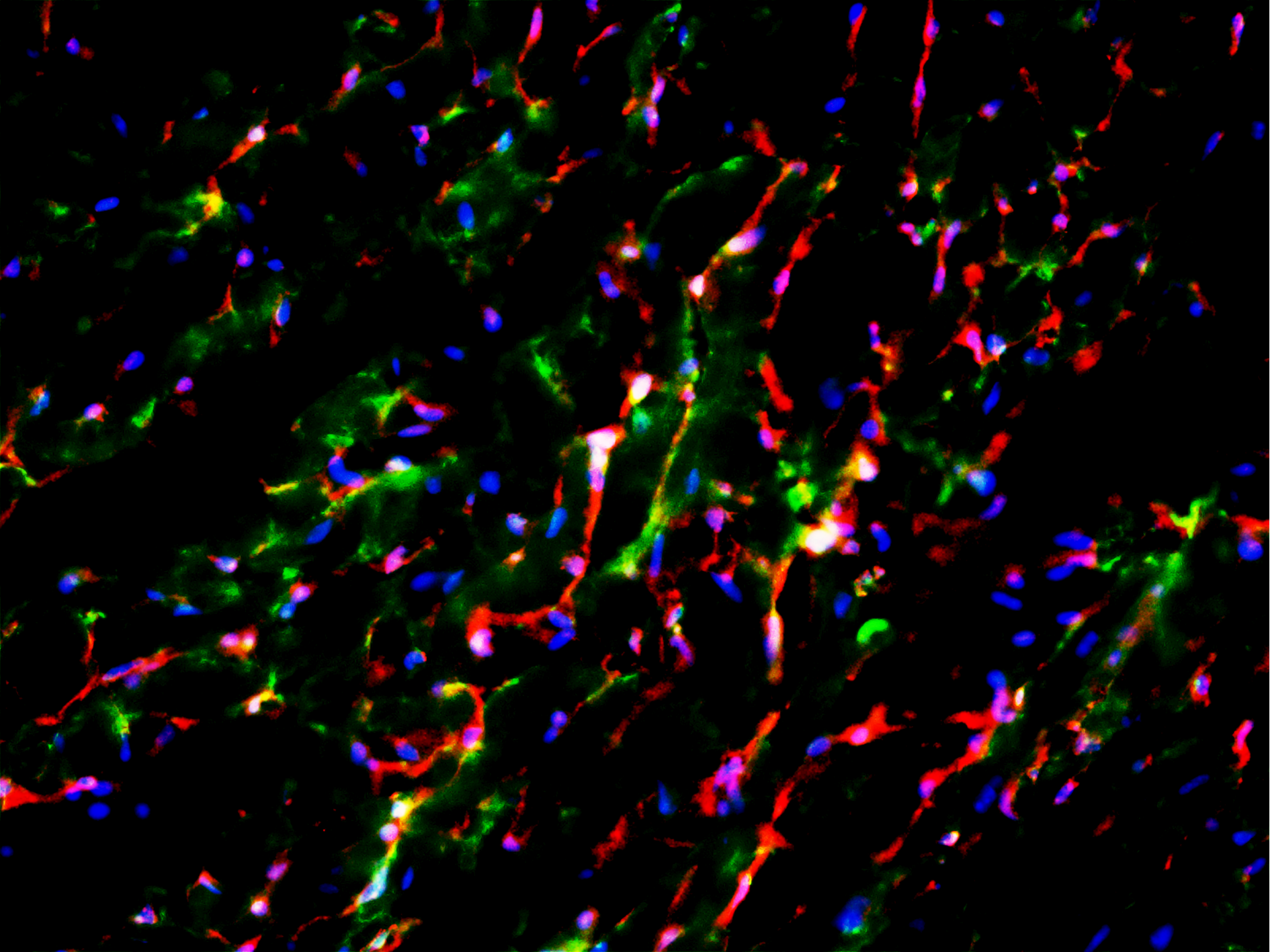

Supplement: Supplementary file 3 — Supplementary Information 3. [file 41598_2024_57595_MOESM3_ESM.zip › Raw figure/Figure2E/Nuciferine/╬┤▒Ω╠Γ-224.tif]

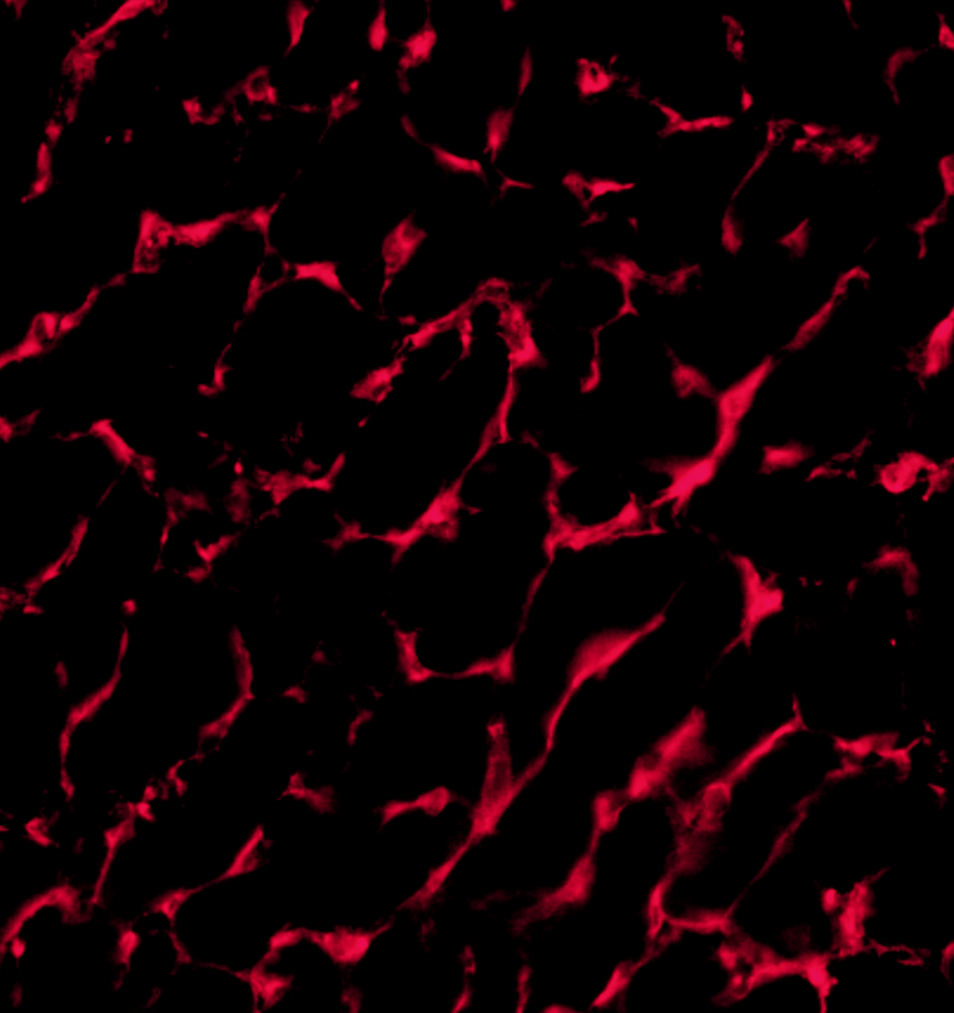

Supplement: Supplementary file 3 — Supplementary Information 3. [file 41598_2024_57595_MOESM3_ESM.zip › Raw figure/Figure2E/Sham/452.tif]

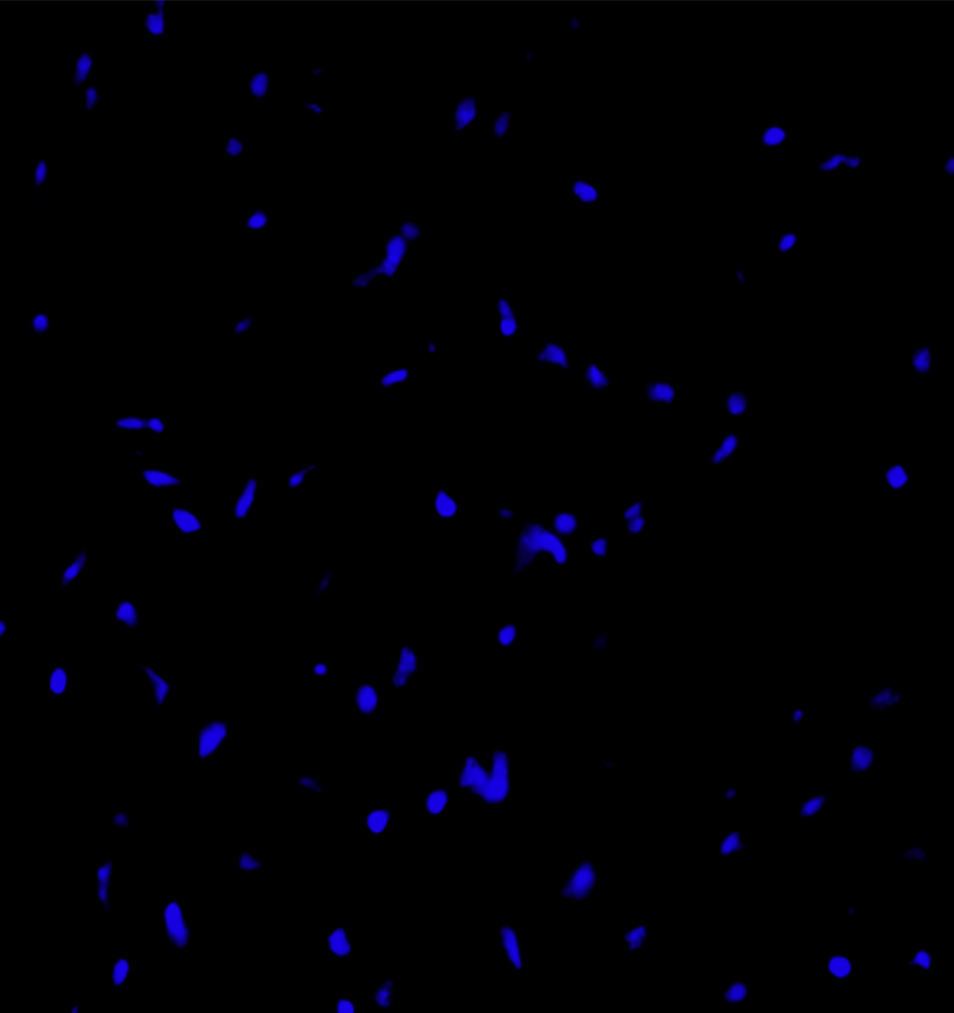

Supplement: Supplementary file 3 — Supplementary Information 3. [file 41598_2024_57595_MOESM3_ESM.zip › Raw figure/Figure2E/Sham/452747.tif]

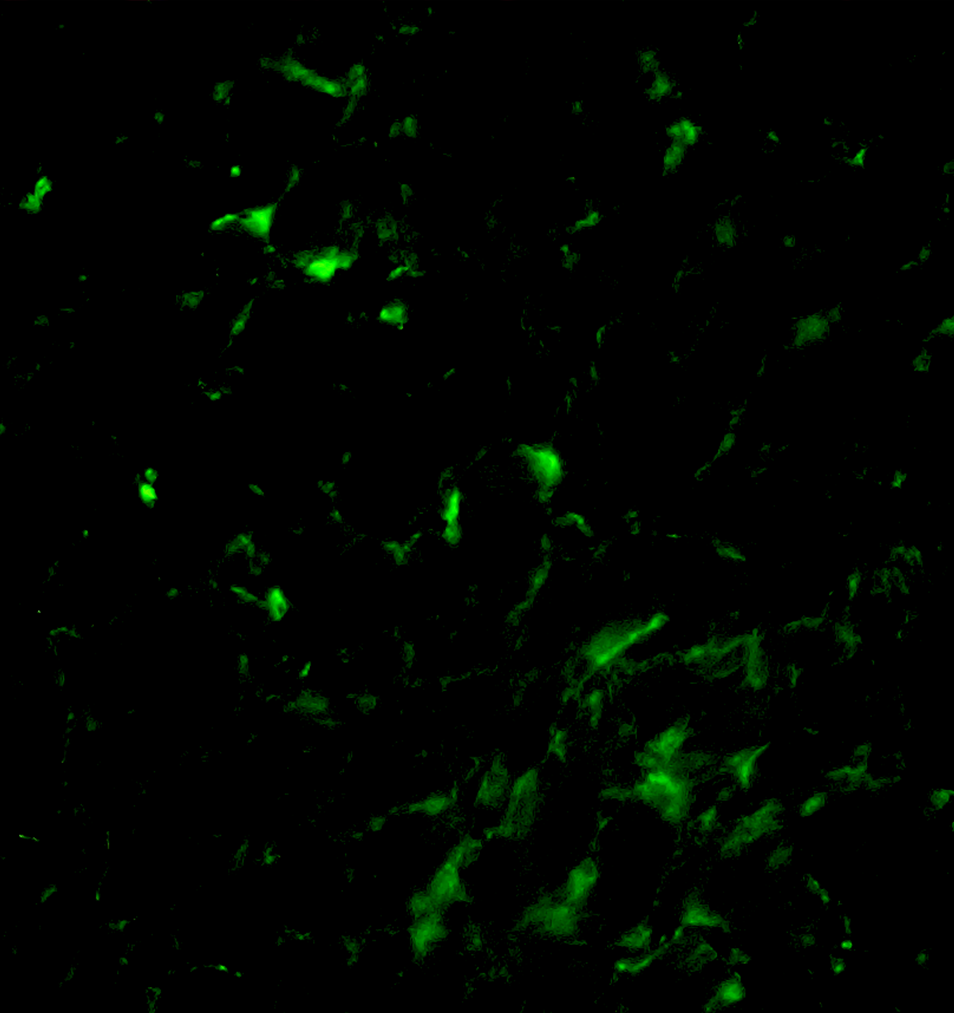

Supplement: Supplementary file 3 — Supplementary Information 3. [file 41598_2024_57595_MOESM3_ESM.zip › Raw figure/Figure2E/Sham/856.tif]

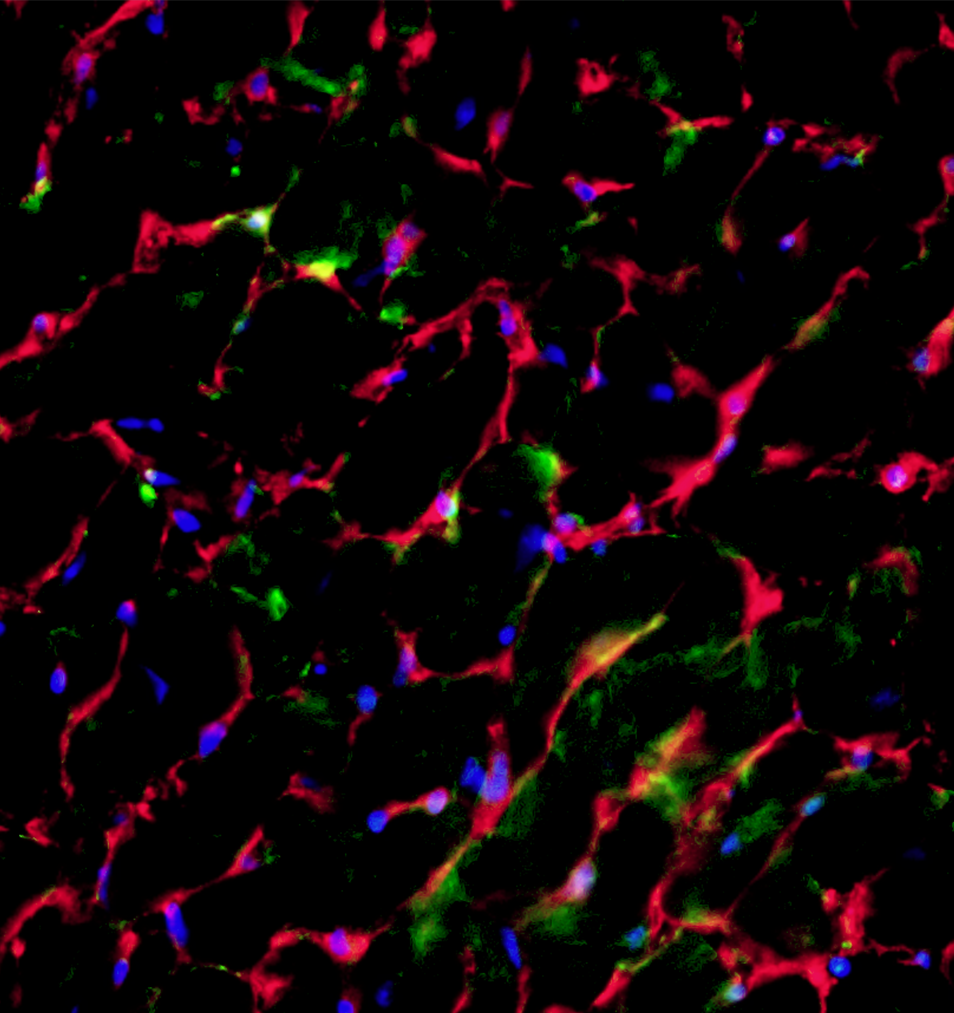

Supplement: Supplementary file 3 — Supplementary Information 3. [file 41598_2024_57595_MOESM3_ESM.zip › Raw figure/Figure2E/Sham/╬┤▒Ω╠Γ-2.tif]

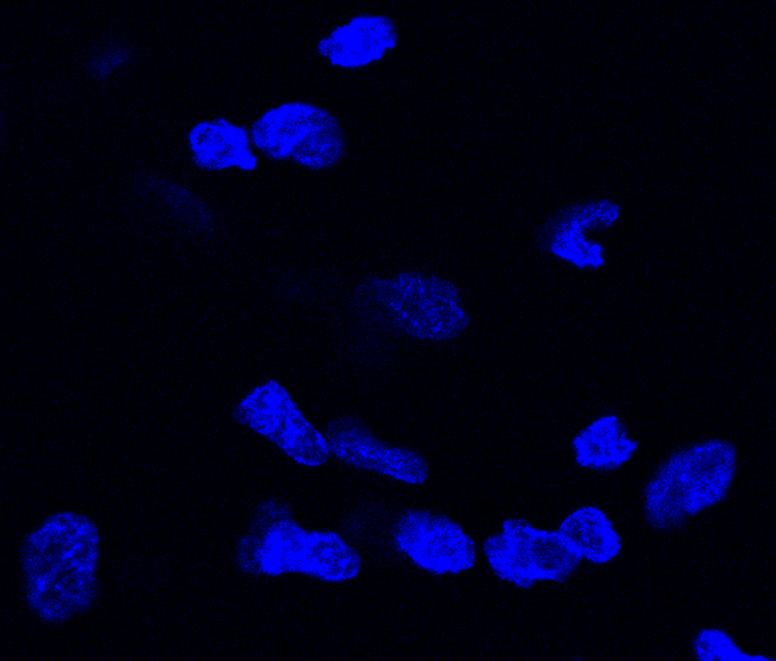

Supplement: Supplementary file 3 — Supplementary Information 3. [file 41598_2024_57595_MOESM3_ESM.zip › Raw figure/Figure4D/Ctrl/DAPI.tif]

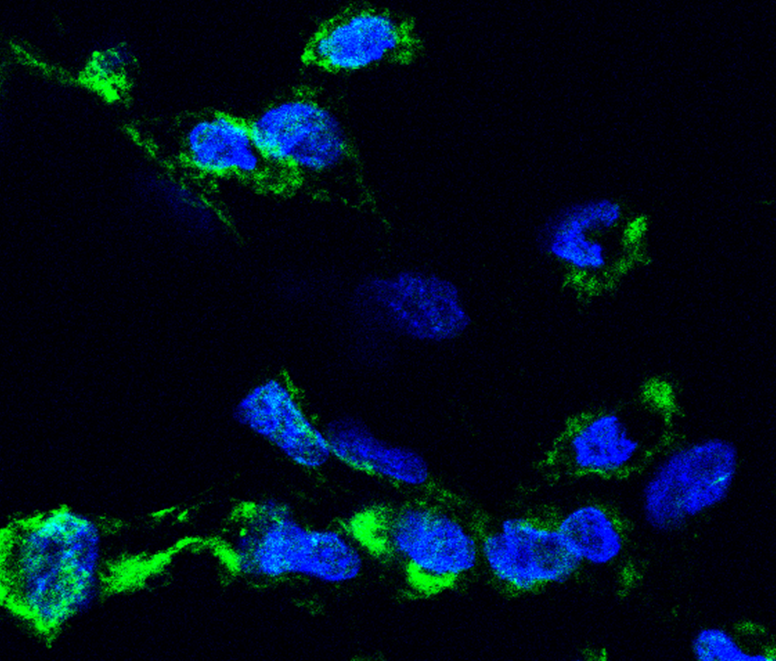

Supplement: Supplementary file 3 — Supplementary Information 3. [file 41598_2024_57595_MOESM3_ESM.zip › Raw figure/Figure4D/Ctrl/Merge.tif]

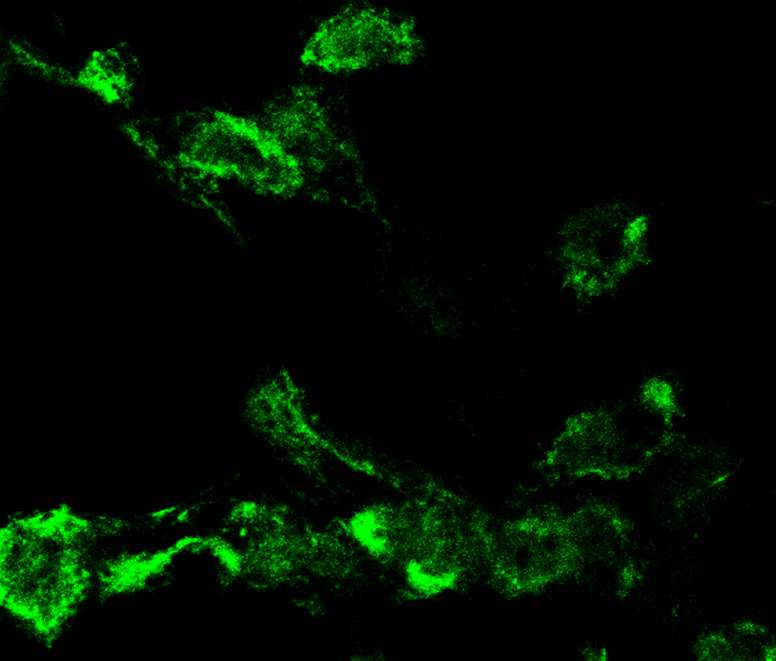

Supplement: Supplementary file 3 — Supplementary Information 3. [file 41598_2024_57595_MOESM3_ESM.zip › Raw figure/Figure4D/Ctrl/VE-cadherin.tif]

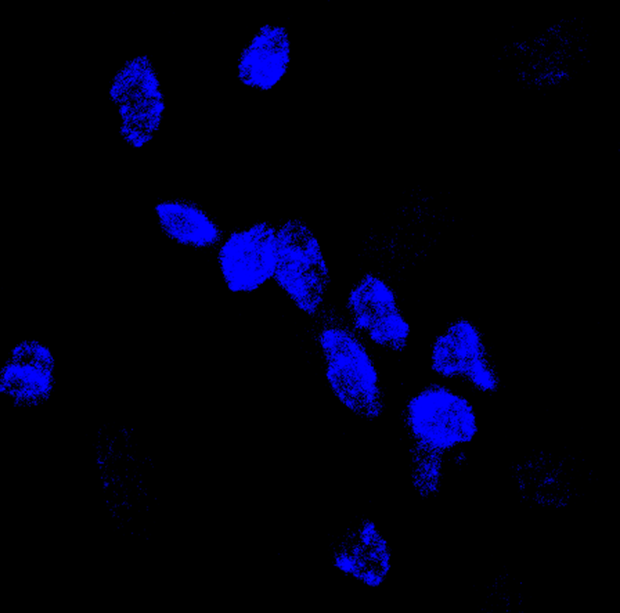

Supplement: Supplementary file 3 — Supplementary Information 3. [file 41598_2024_57595_MOESM3_ESM.zip › Raw figure/Figure4D/DMSO/DAPI.tif]

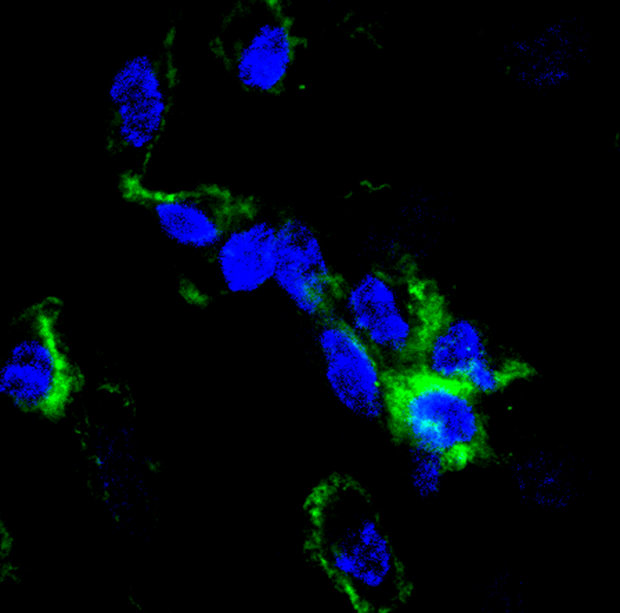

Supplement: Supplementary file 3 — Supplementary Information 3. [file 41598_2024_57595_MOESM3_ESM.zip › Raw figure/Figure4D/DMSO/merge.tif]

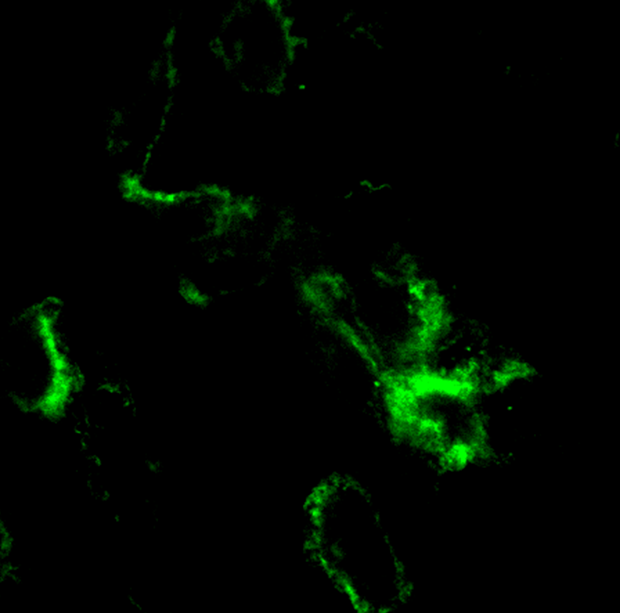

Supplement: Supplementary file 3 — Supplementary Information 3. [file 41598_2024_57595_MOESM3_ESM.zip › Raw figure/Figure4D/DMSO/Ve-cadherin.tif]

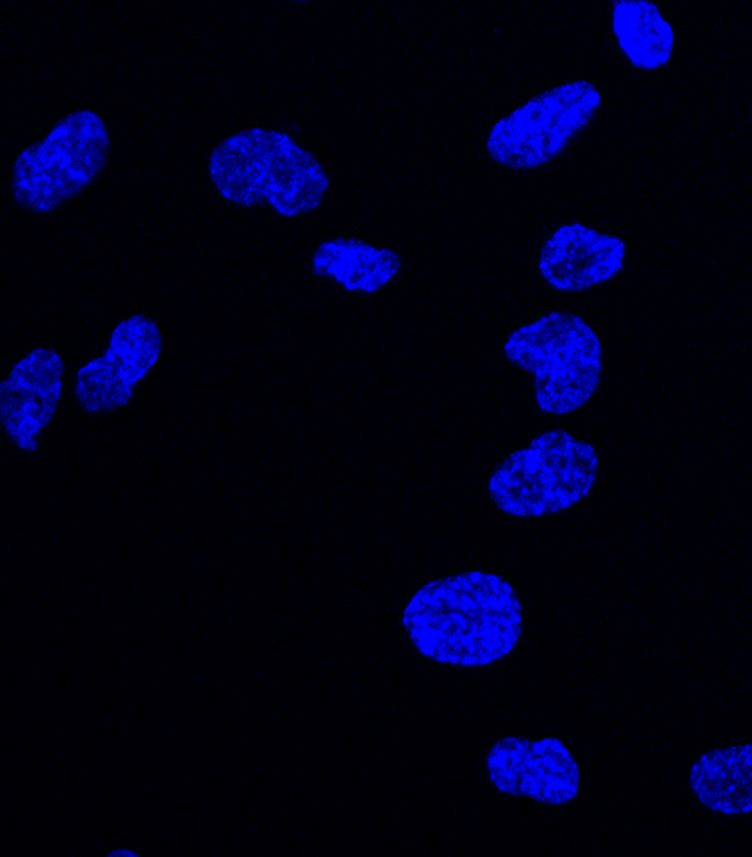

Supplement: Supplementary file 3 — Supplementary Information 3. [file 41598_2024_57595_MOESM3_ESM.zip › Raw figure/Figure4D/Normal/DAPI.tif]

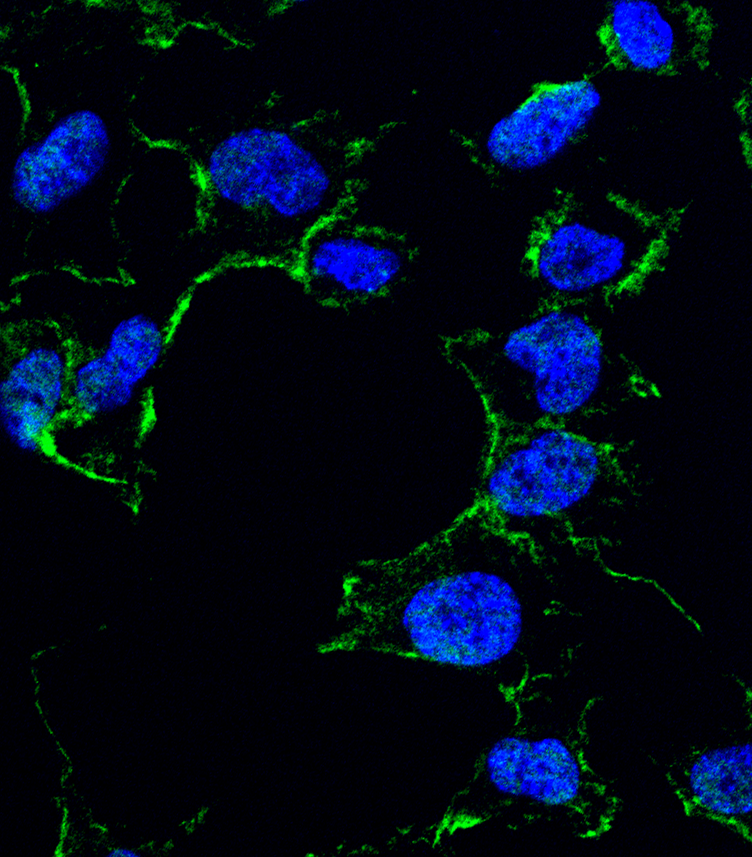

Supplement: Supplementary file 3 — Supplementary Information 3. [file 41598_2024_57595_MOESM3_ESM.zip › Raw figure/Figure4D/Normal/merge.tif]

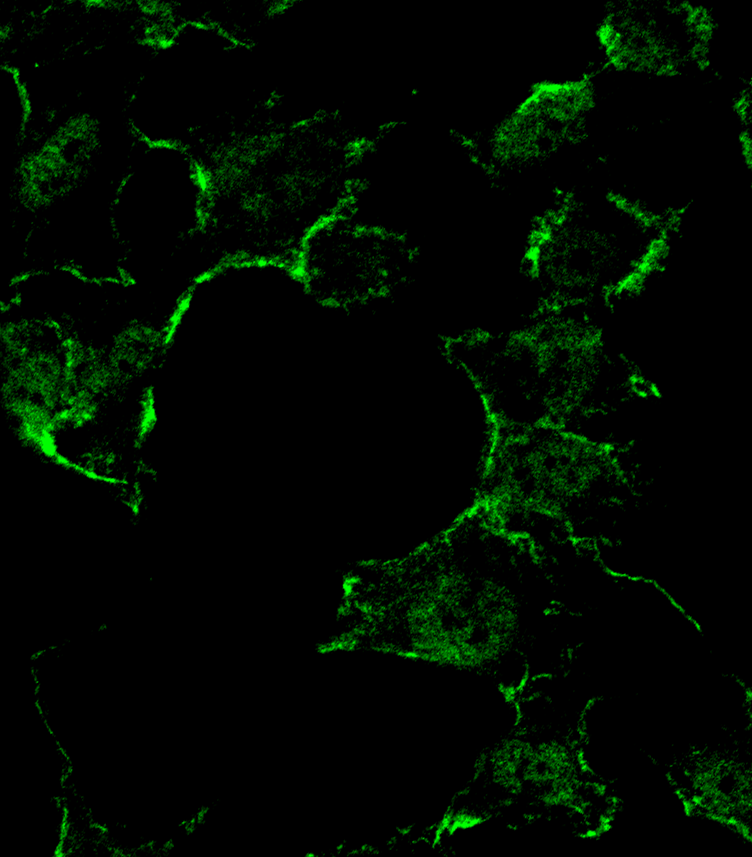

Supplement: Supplementary file 3 — Supplementary Information 3. [file 41598_2024_57595_MOESM3_ESM.zip › Raw figure/Figure4D/Normal/Ve-cadherin.tif]

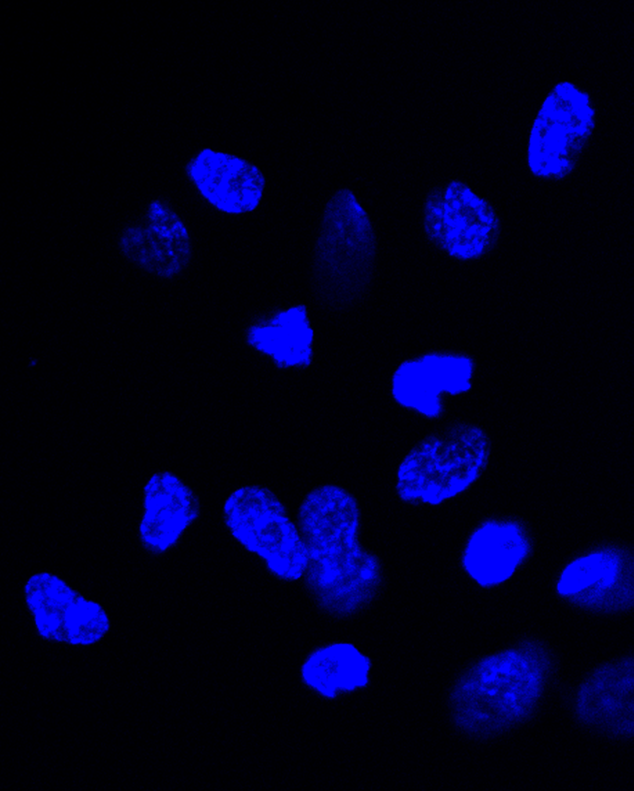

Supplement: Supplementary file 3 — Supplementary Information 3. [file 41598_2024_57595_MOESM3_ESM.zip › Raw figure/Figure4D/Nuciferine/DAPI.tif]

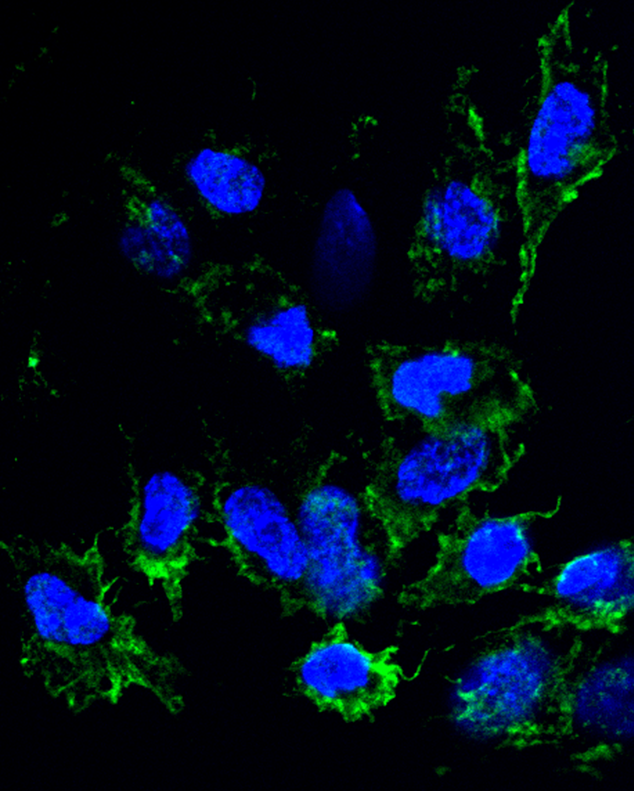

Supplement: Supplementary file 3 — Supplementary Information 3. [file 41598_2024_57595_MOESM3_ESM.zip › Raw figure/Figure4D/Nuciferine/merge.tif]

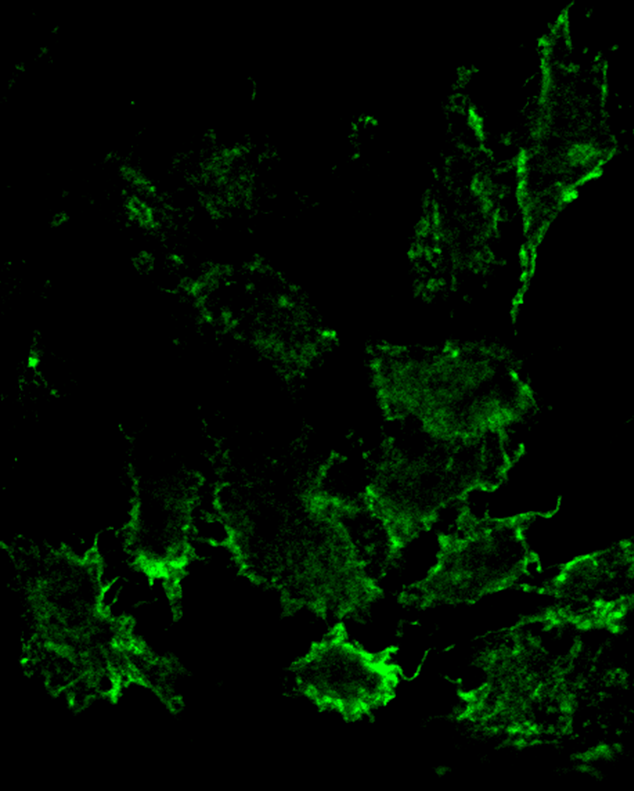

Supplement: Supplementary file 3 — Supplementary Information 3. [file 41598_2024_57595_MOESM3_ESM.zip › Raw figure/Figure4D/Nuciferine/Ve-cadherin.tif]

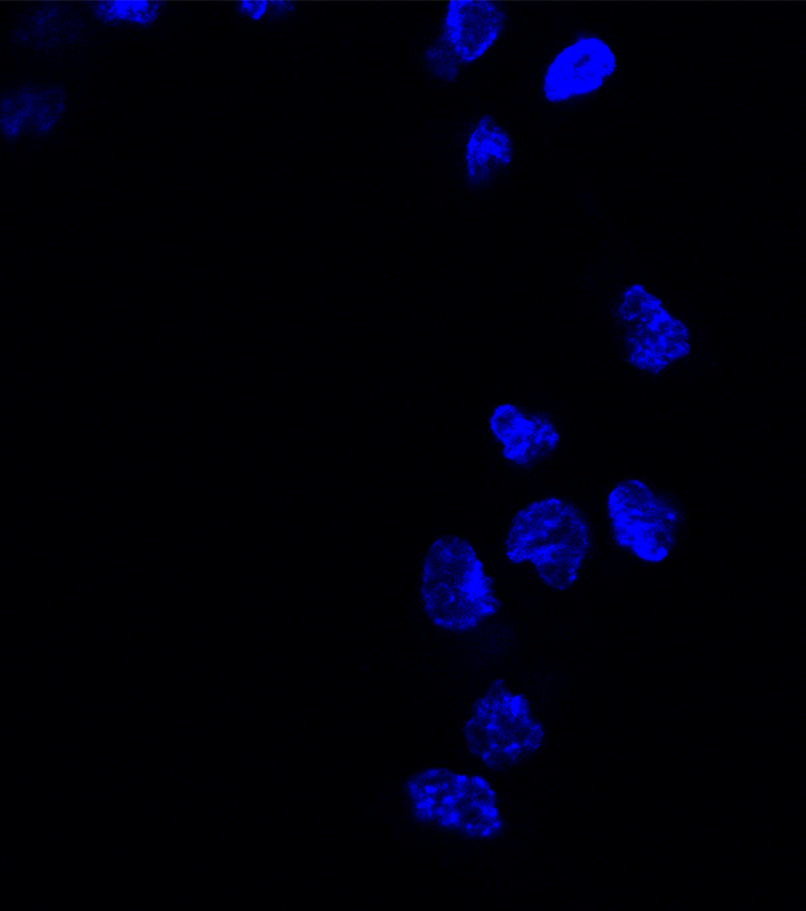

Supplement: Supplementary file 3 — Supplementary Information 3. [file 41598_2024_57595_MOESM3_ESM.zip › Raw figure/Figure6E/Ctrl/DAPI.tif]

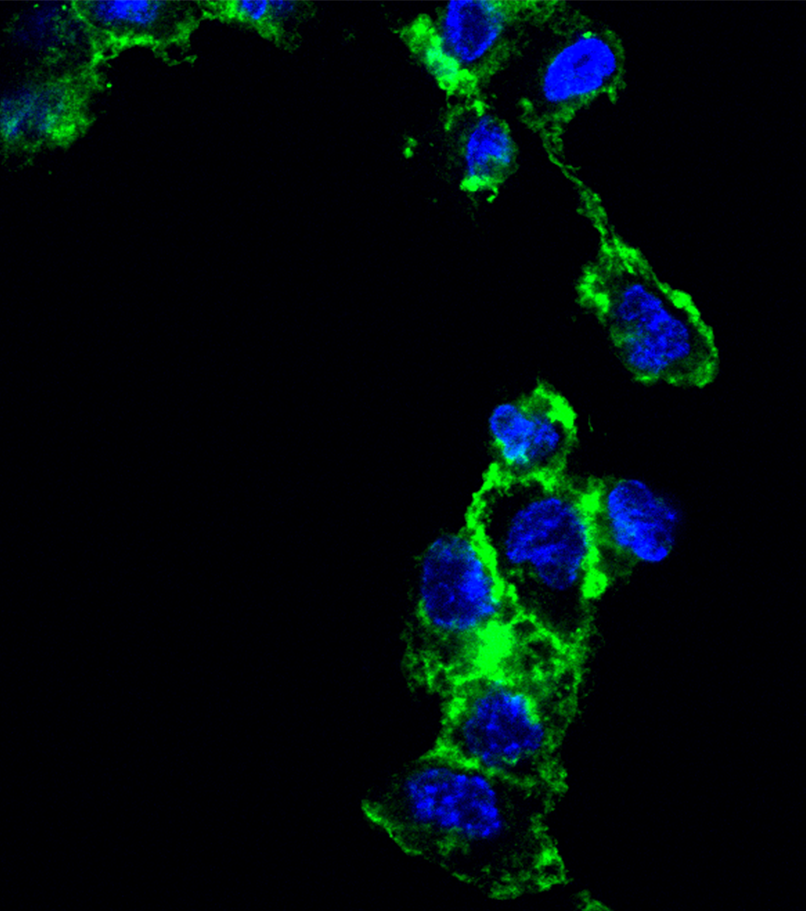

Supplement: Supplementary file 3 — Supplementary Information 3. [file 41598_2024_57595_MOESM3_ESM.zip › Raw figure/Figure6E/Ctrl/merge.tif]

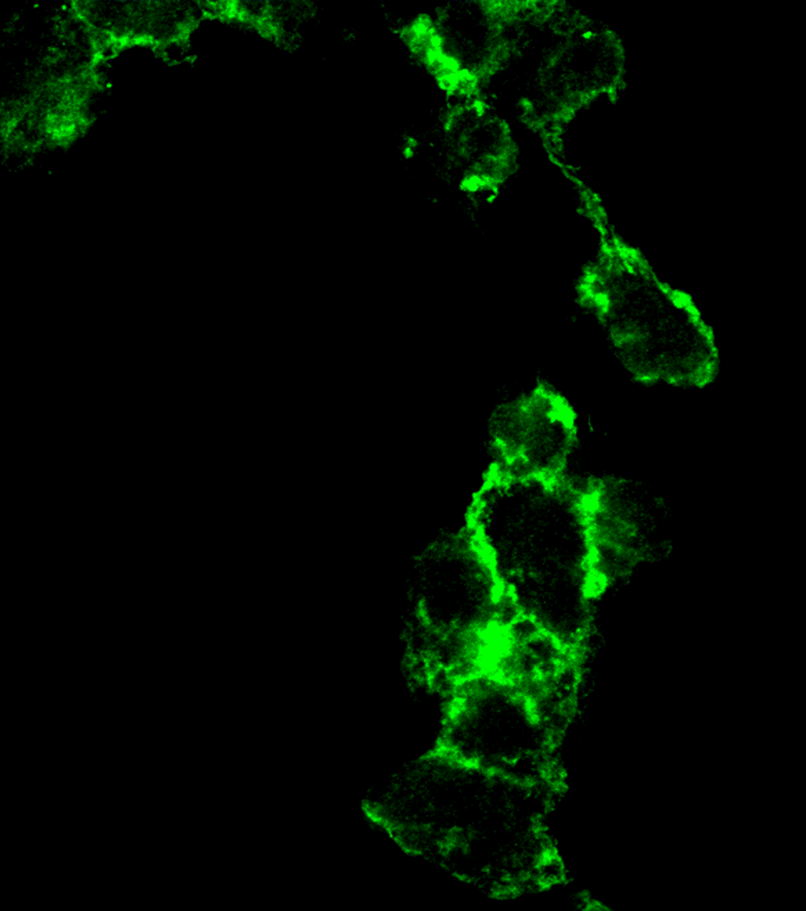

Supplement: Supplementary file 3 — Supplementary Information 3. [file 41598_2024_57595_MOESM3_ESM.zip › Raw figure/Figure6E/Ctrl/Ve-cadherin.tif]

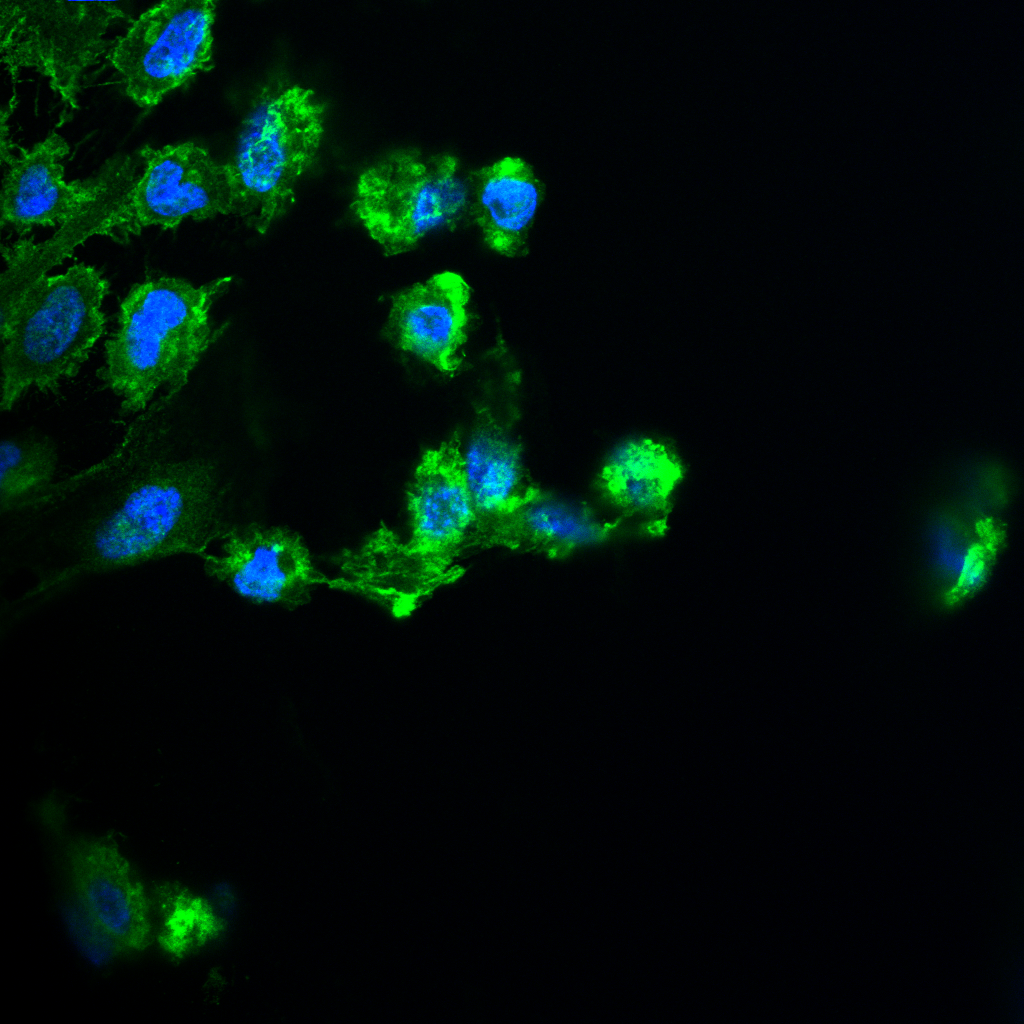

Supplement: Supplementary file 3 — Supplementary Information 3. [file 41598_2024_57595_MOESM3_ESM.zip › Raw figure/Figure6E/DMSO/DAPI.tif]

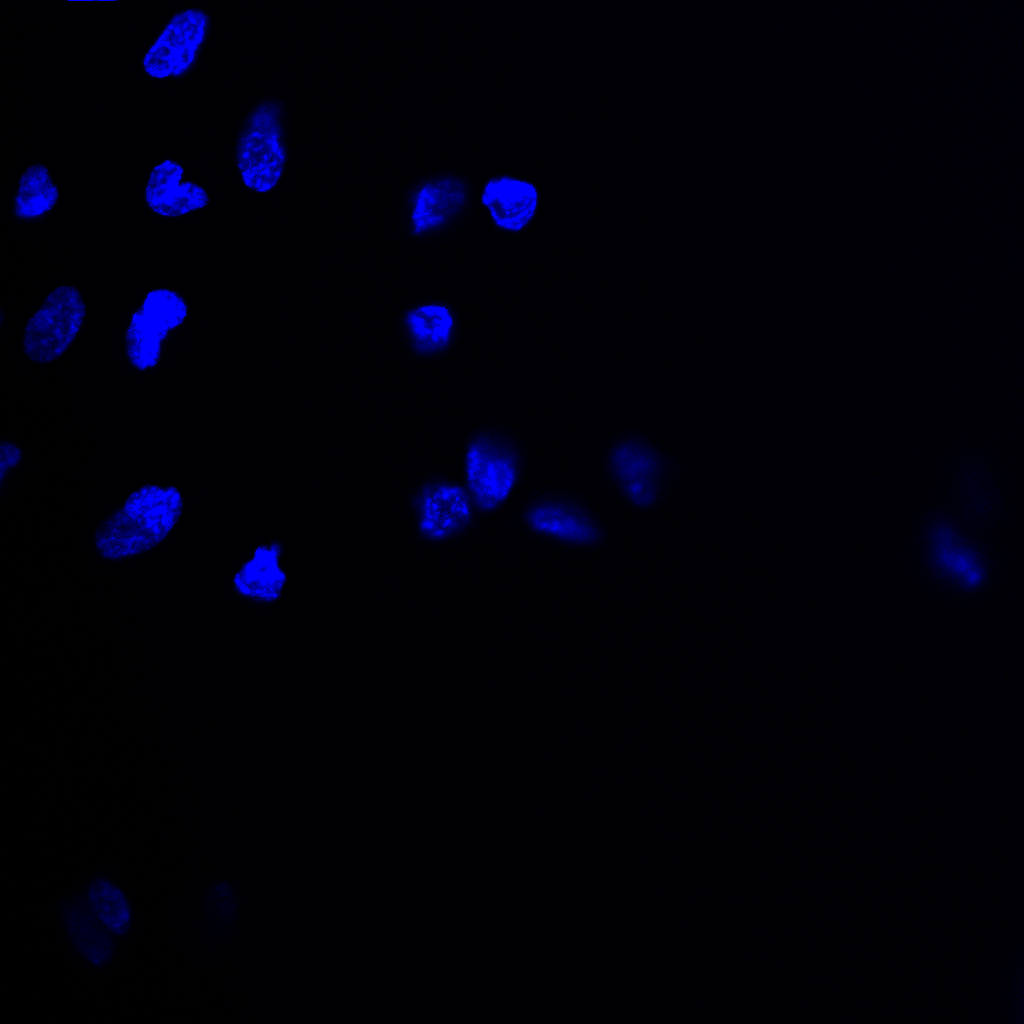

Supplement: Supplementary file 3 — Supplementary Information 3. [file 41598_2024_57595_MOESM3_ESM.zip › Raw figure/Figure6E/DMSO/merge.tif]

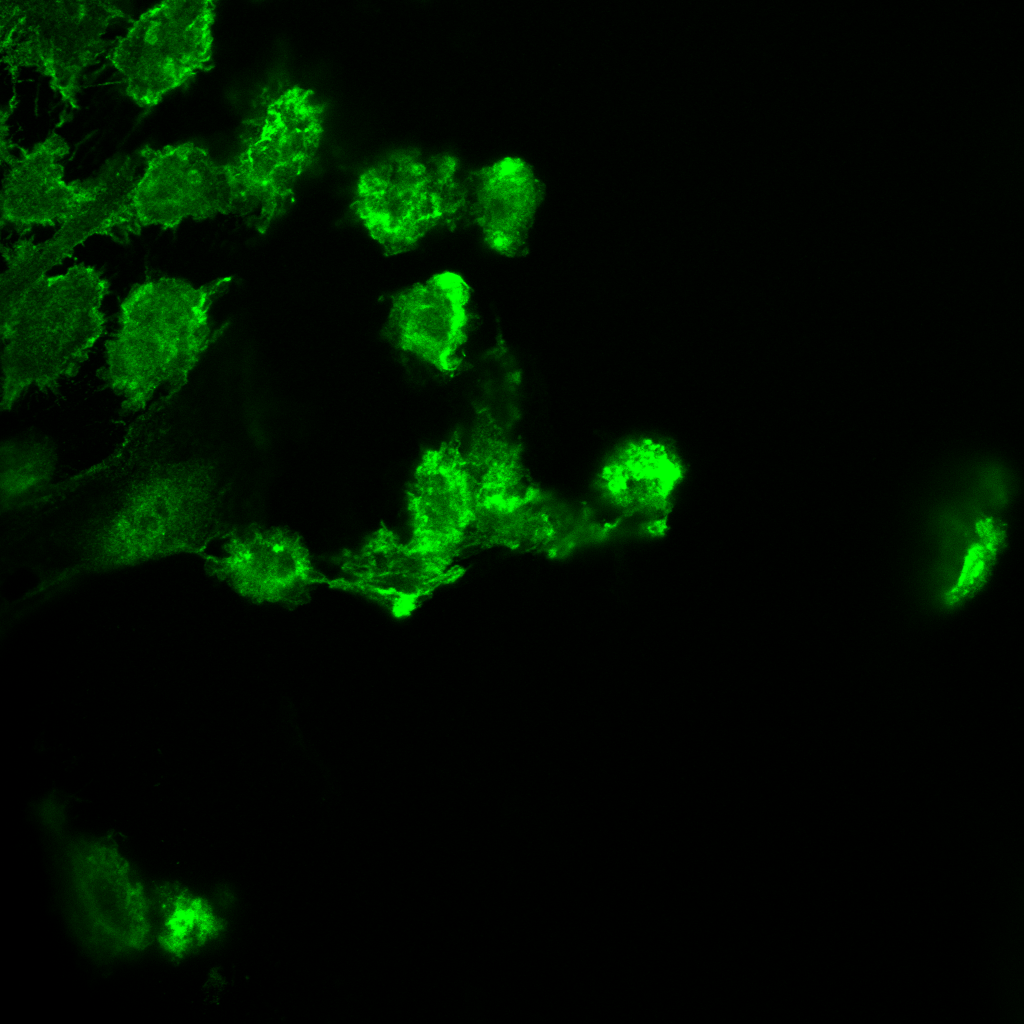

Supplement: Supplementary file 3 — Supplementary Information 3. [file 41598_2024_57595_MOESM3_ESM.zip › Raw figure/Figure6E/DMSO/Ve-cadherin.tif]

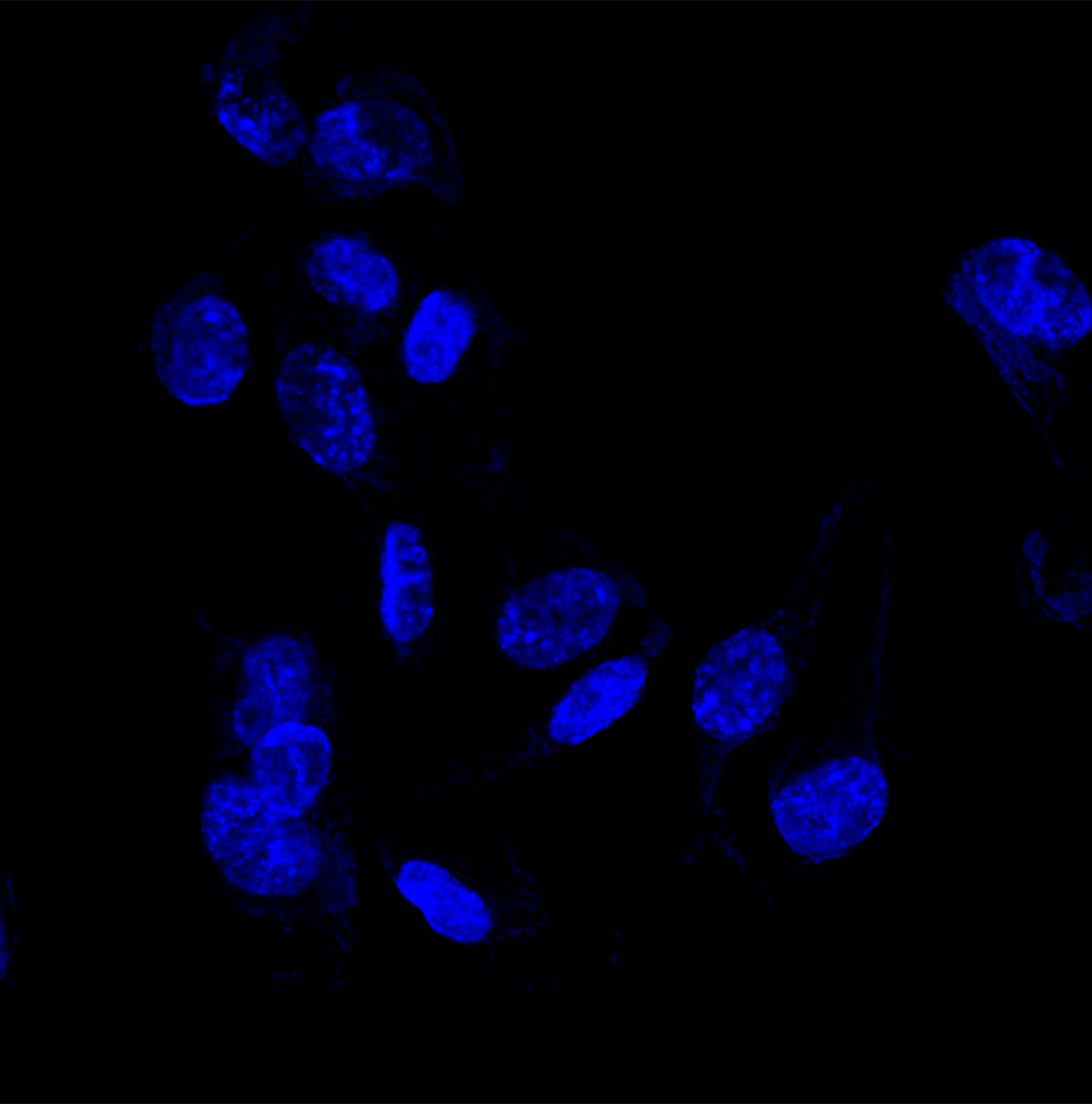

Supplement: Supplementary file 3 — Supplementary Information 3. [file 41598_2024_57595_MOESM3_ESM.zip › Raw figure/Figure6E/N+wortmannin/DAPI.tif]

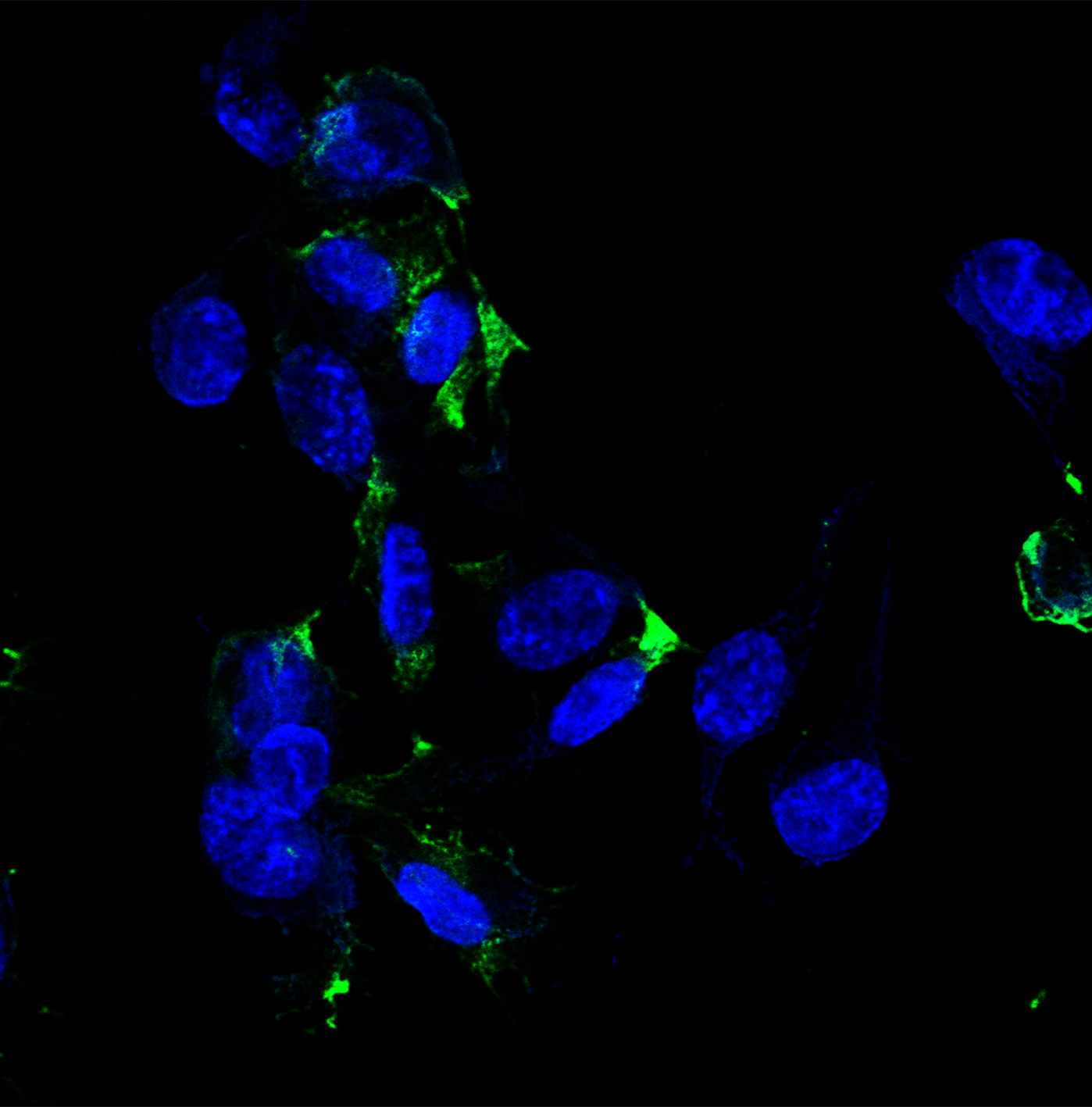

Supplement: Supplementary file 3 — Supplementary Information 3. [file 41598_2024_57595_MOESM3_ESM.zip › Raw figure/Figure6E/N+wortmannin/merge.tif]

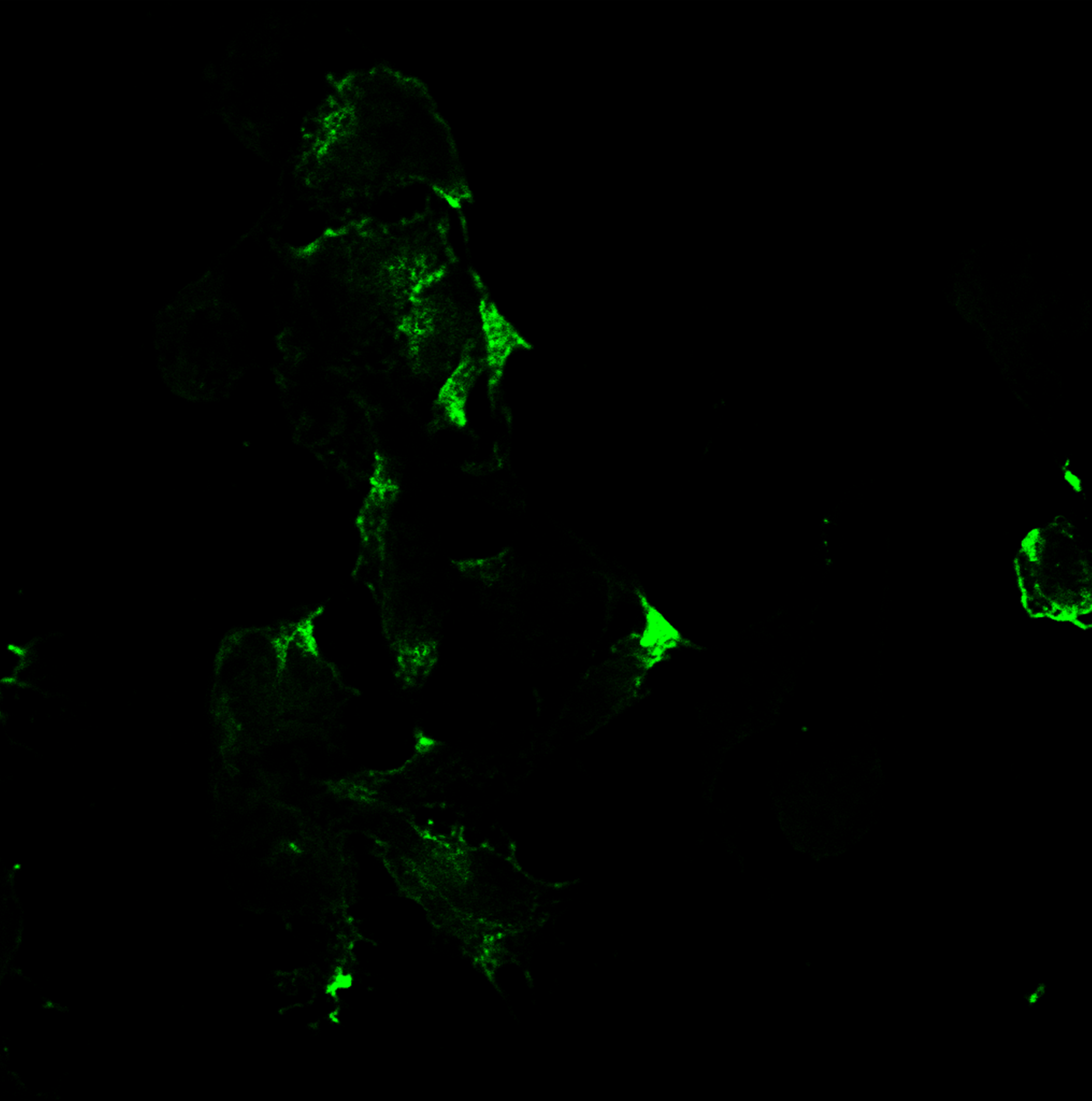

Supplement: Supplementary file 3 — Supplementary Information 3. [file 41598_2024_57595_MOESM3_ESM.zip › Raw figure/Figure6E/N+wortmannin/Ve-cadherin.tif]

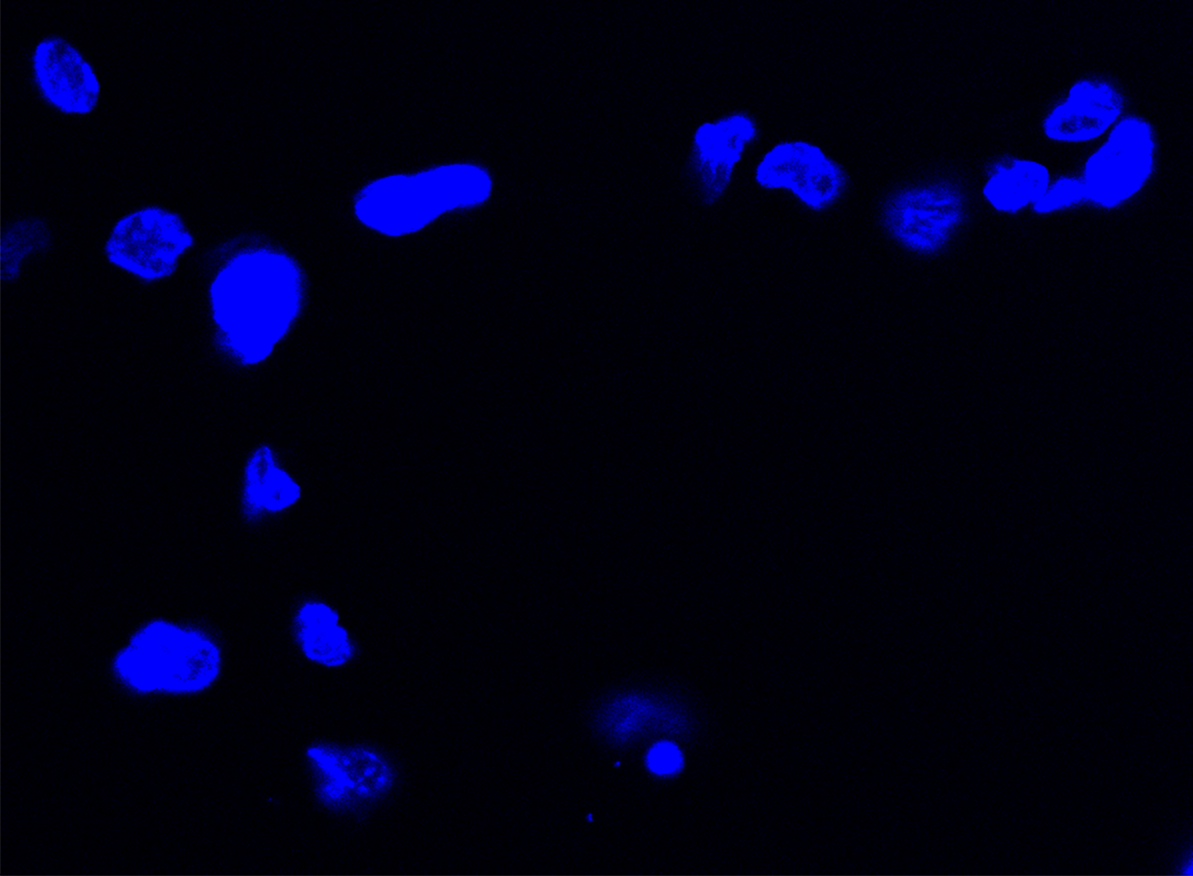

Supplement: Supplementary file 3 — Supplementary Information 3. [file 41598_2024_57595_MOESM3_ESM.zip › Raw figure/Figure6E/Normal/DAPI.tif]

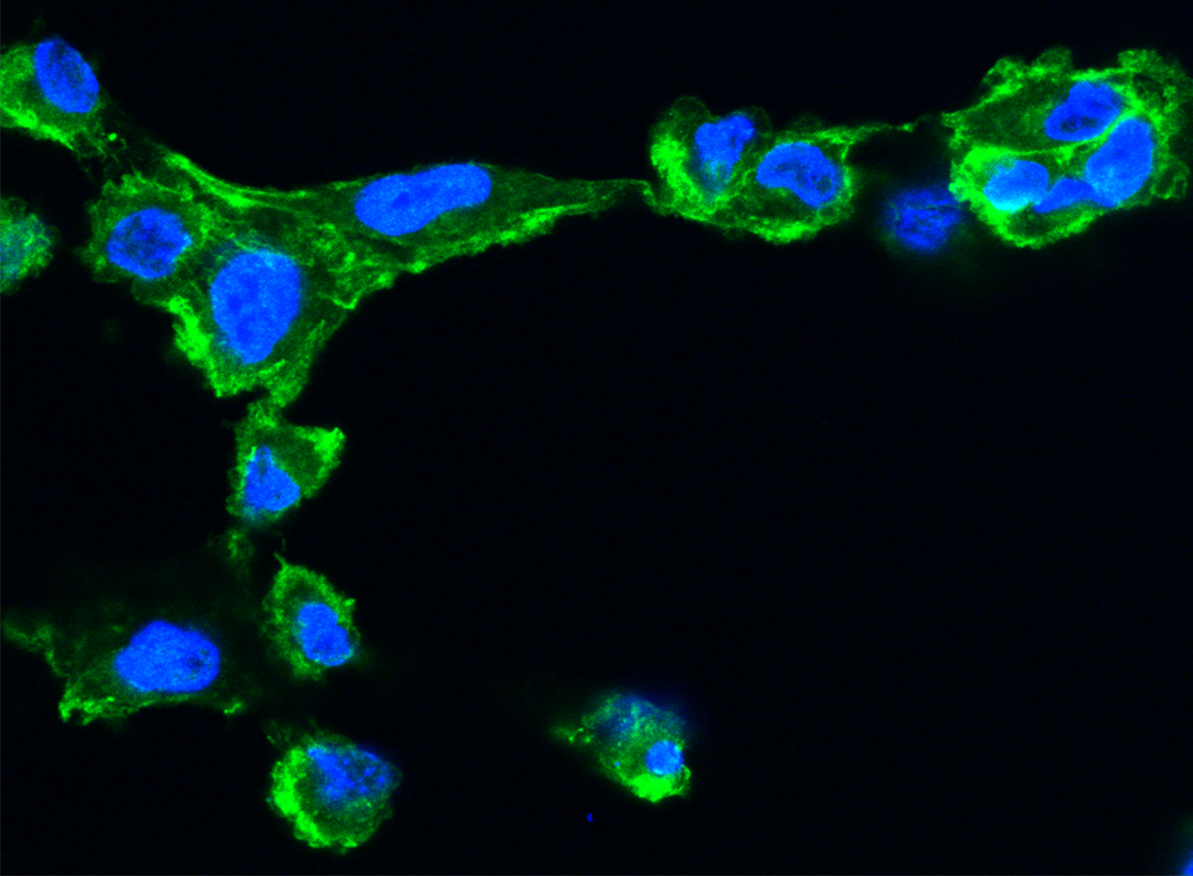

Supplement: Supplementary file 3 — Supplementary Information 3. [file 41598_2024_57595_MOESM3_ESM.zip › Raw figure/Figure6E/Normal/merge.tif]

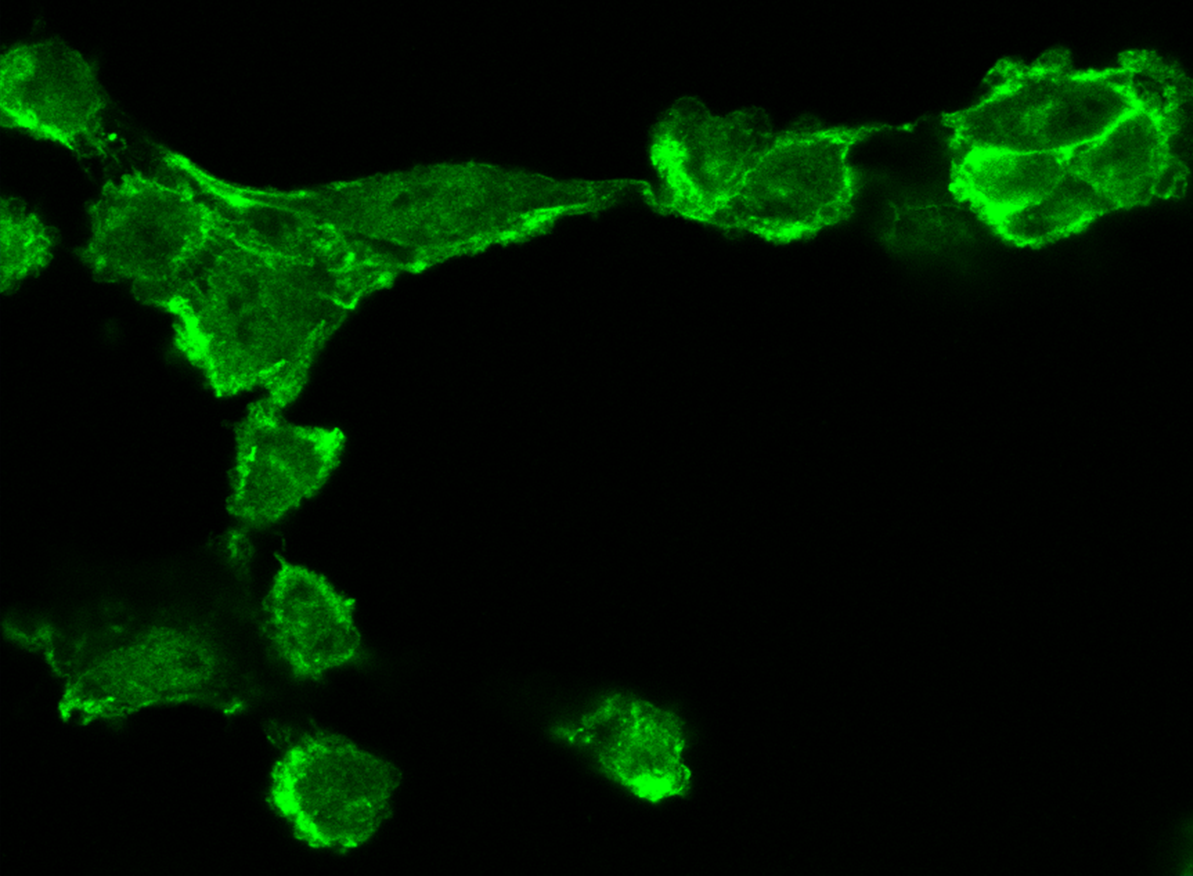

Supplement: Supplementary file 3 — Supplementary Information 3. [file 41598_2024_57595_MOESM3_ESM.zip › Raw figure/Figure6E/Normal/Ve-cadherin.tif]

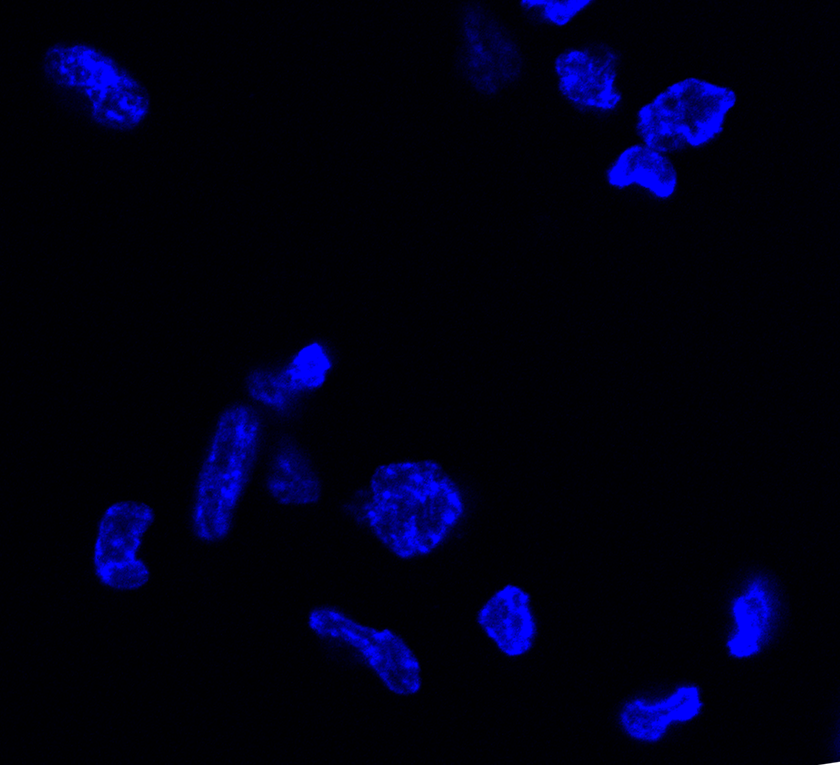

Supplement: Supplementary file 3 — Supplementary Information 3. [file 41598_2024_57595_MOESM3_ESM.zip › Raw figure/Figure6E/Nuciferine/DAPI.tif]

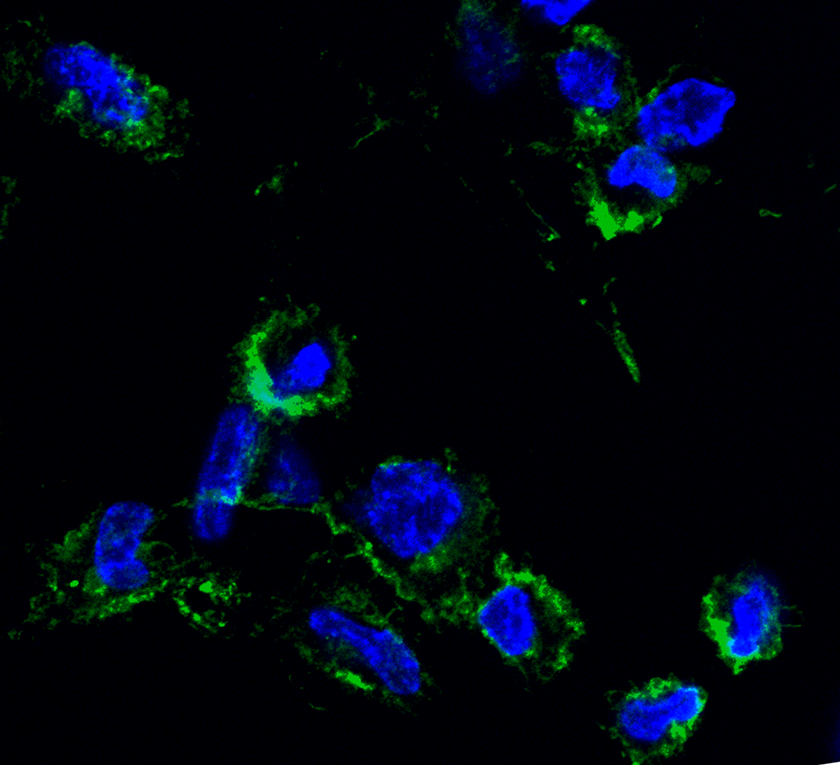

Supplement: Supplementary file 3 — Supplementary Information 3. [file 41598_2024_57595_MOESM3_ESM.zip › Raw figure/Figure6E/Nuciferine/merge.tif]

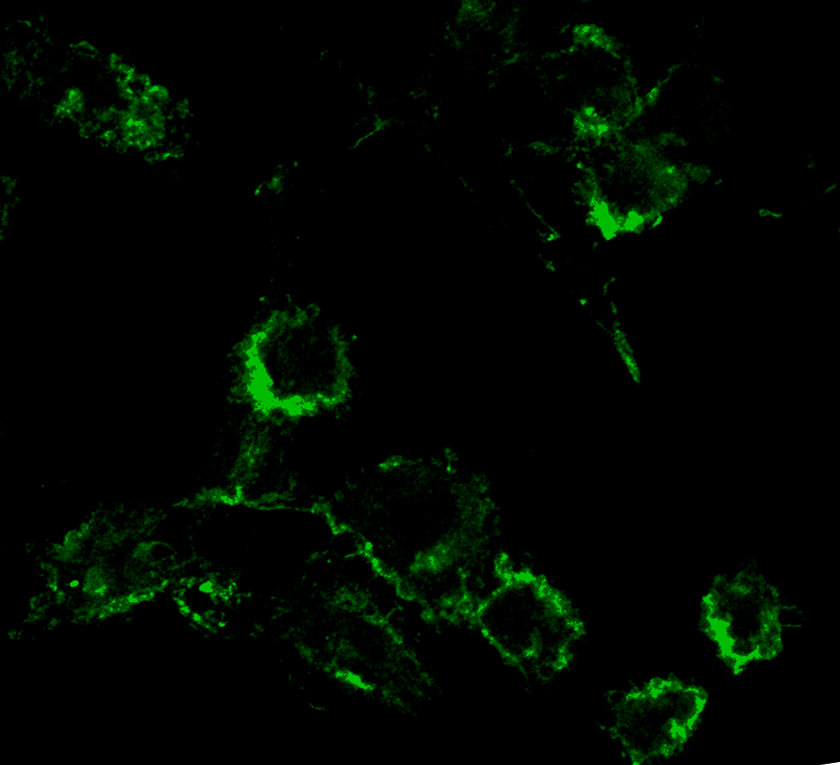

Supplement: Supplementary file 3 — Supplementary Information 3. [file 41598_2024_57595_MOESM3_ESM.zip › Raw figure/Figure6E/Nuciferine/Ve-cadherin.tif]
